# Effects of combined aerobic exercise and diet on cardiometabolic health in patients with obesity and type 2 diabetes: a systematic review and meta-analysis

**DOI:** 10.1186/s13102-023-00766-5

**Published:** 2023-12-04

**Authors:** Sameer Badri Al-Mhanna, Sílvia Rocha-Rodriguesc, Mahaneem Mohamed, Alexios Batrakoulis, Monira I. Aldhahi, Hafeez Abiola Afolabi, Fatma Hilal Yagin, Maha H. Alhussain, Mehmet Gülü, Bishir Daku Abubakar, Wan Syaheedah Wan Ghazali, Abdullah F. Alghannam, Georgian Badicu

**Affiliations:** 1 Center for Global Health Research, Saveetha Medical College and Hospitals, Saveetha Institute of Medical and Technical Sciences, Saveetha University, New Delhi, India; 2 Department of Physiology, School of Medical Sciences, Universiti Sains Malaysia, Kubang Kerian, Kelantan 16150 Malaysia; 3 Escola Superior Desporto e Lazer, Instituto Politécnico de Viana do Castelo, Viana do Castelo, Portugal; 4 Tumour & Microenvironment Interactions Group, INEB- Institute of Biomedical Engineering, i3S-Instituto de Investigação e Inovação em Saúde, Universidade do Porto, Porto, Portugal; 5 Department of Physical Education and Sport Science, School of Physical Education, Sport Science and Dietetics, University of Thessaly, Trikala, Greece; 6 Department of Rehabilitation, College of Health and Rehabilitation Sciences, Princess Nourah bint Abdulrahman University, Riyadh, Saudi Arabia; 7 Department of General Surgery, School of Medical Sciences, Universiti Sains Malaysia, Kubang Kerian, Kelantan Malaysia; 8 Department of Biostatistics and Medical Informatics, Faculty of Medicine, Inonu University, Malatya, Turkey; 9 Department of Food Science and Nutrition, College of Food and Agricultural Science, King Saud University, Riyadh, Saudi Arabia; 10 Department of Sports Management, Faculty of Sport Sciences, Kirikkale University, Kirikkale, Turkey; 11 Department of Human Physiology, Federal University Dutse, Dutse, Jigawa State Nigeria; 12 Lifestyle and Health Research Center, Health Sciences Research Center, Princess Nourah bint Abdulrahman University, Riyadh, Saudi Arabia; 13 Department of Physical Education and Special Motricity, Transilvania University of Brasov, Brasov, Romania

**Keywords:** Physical activity, Exercise, Overweight, Metabolic syndrome, Lifestyle intervention

## Abstract

**Background:**

Lifestyle modifications involving diet and exercise are recommended for patients diagnosed with obesity and type 2 diabetes mellitus (T2DM). The purpose of this review was to systematically evaluate the effects of combined aerobic exercise and diet (AEDT) on various cardiometabolic health-related indicators among individuals with obesity and T2DM.

**Methodology:**

A comprehensive search of the PubMed/Medline, Web of Science, Scopus, Science Direct, Cochrane, and Google Scholar databases was conducted for this meta-analysis. The Cochrane risk of bias tool was used to evaluate eligible studies, and the GRADE tool was used to rate the certainty of evidence. A random-effects model for continuous variables was used, and the results were presented as mean differences or standardised mean differences with 95% confidence intervals.

**Results:**

A total of 16,129 studies were retrieved; 20 studies were included, and data were extracted from 1,192 participants. The findings revealed significant improvements in body mass index, body weight, waist circumference, systolic blood pressure, diastolic blood pressure, total cholesterol, triglycerides, fasting blood glucose, fasting plasma insulin, glycated hemoglobin, leptin, interleukin-6, C-reactive protein, and adiponectin *(p* < 0.05) compared to the standard treatment (ST) group. No significant differences were observed between the AEDT and ST groups in fat mass, hip circumference, waist-to-hip ratio, high-density lipoprotein cholesterol, low-density lipoprotein cholesterol, and tumor necrosis factor-alpha. The present findings are based on low- to moderate-quality evidence.

**Conclusions:**

AEDT may be a critical behavior for holistic cardiometabolic health-related benefits as a contemporary anti-obesity medication due to its significant positive impact on patients with obesity and T2DM. Nevertheless, further robust evidence is necessary to determine whether AEDT is an effective intervention for lowering cardiovascular and metabolic risk factors among individuals with obesity and T2DM.

**Supplementary Information:**

The online version contains supplementary material available at 10.1186/s13102-023-00766-5.

## Introduction

The prevalence of obesity and type 2 diabetes mellitus (T2DM) continue to escalate, largely attributed to the effects of urbanisation, which, in turn, results in notably reduced levels of physical activity [1]. Studies found that people living in urban areas have higher body mass index (BMI) than those living in rural areas [2, 3]. The incidence of these two pathologies is estimated to double in the following decades [4]. With increasing urbanisation and the abundance of high-calorie meals, there is a noticeable change in the expression and regulation of genes related to metabolic pathways, energy consumption, and body weight management [5]. These genetic and environmental factors, such as obesity, poor diet, and sedentarism strongly contribute to multiple pathophysiological changes that lead to adversely impaired glucose metabolism in T2DM [6]. Some genome-wide association epidemiological relationship studies conducted associate obesity and other metabolic diseases with around 97 loci of body mass index (BMI) variants linked to relevant cardiometabolic traits, influencing metabolic pathways probably by shared genetic effects and cross-phenotypic correlations [7].

Studies evaluating specific diet regimens for T2DM found that all diet types are designed to improve metabolic imbalances. Nonetheless, the responses of individuals vary according to their needs and pathophysiological attributes, resulting in a deficiency of personalised dietary plans [8]. A comparable tendency was observed in Krebs, Elley [9] randomised controlled trial (RCT), demonstrating a modest reduction in weight and waist circumference. Also, factors influencing nutrient utilisation and metabolism encompass the composition and activity of the gut microbiota [10], which collectively contribute to an individual’s response to specific diets and impact the body’s energy expenditure.

A lack of physical exercise is a risk factor per se comparable to having a previous cardiovascular event and even a greater predictor of all-cause mortality in individuals with obesity, than hypertension, smoking status, or cholesterol levels [11]. Although physical exercise improves vascular structure and endothelial function, lowers blood pressure and lipid levels, improves glycemic control [12], and reduces chronic inflammation and body mass [13], people with excessive weight demonstrate exercise motivation due to body dissatisfaction [14]. In T2DM patients, increased glycated haemoglobin (HbA1c) levels are predictive of vascular complications, and HbA1c was reported to reduce in direct correlation with exercise intensity. Although there is currently no known cure for T2DM, treatment options are centred on lifestyle changes, managing obesity, insulin sensitizers, and oral hypoglycemic drugs, these are still first-line interventions, particularly for obese T2DM patients [15].

It is evident that various exercise types have clinically positive effects on populations with obesity, even without weight loss [16–23]. In particular, aerobic training has been reported as a popular [24] and effective exercise mode for improving several cardiovascular and metabolic risk factors among adults with obesity [23]. However, only a few trials have evaluated the effectiveness of AEDT in overweight/obese patients with T2DM. Hence, the long-term implications of AEDT in T2DM patients remain unclear, and therefore, this review aims to evaluate the effects of AEDT on various cardiometabolic health-related indicators among overweight and obese patients with T2DM.

## Materials and methods

### Protocol registration

This systematic review and meta-analysis were conducted in accordance with the Preferred Reporting Items for Systematic Reviews and Meta-Analyses statement guidelines [25]. The study protocol was registered in the International Prospective Register of Systematic Reviews (ID: CRD42023390330).

### Literature search strategy

Articles were retrieved from PubMed/Medline, Web of Science, Scopus, Science Direct, Cochrane Library, and Google Scholar after a systematic electronic search. Four authors (S.B.A.L., W.S.W.G., A.B.D., and H.A.) employed a combination of keywords and Boolean operators, specifically “OR” and “AND” in conducting an electronic search of literature until October 20, 2023. The keywords utilised were “exercise” OR “training” AND “obesity” AND “diabetes” AND “diet” as stated in Table S1, to retrieve pertinent material. The search strategy used keywords related to the PICOS [(P) Population: T2DM patients with overweight or obesity; (I) Intervention: AEDT; (C) Comparator: standard treatment (ST) (patients who continued their usual lifestyle and did not engage in any exercise or diet regimen); (O) Primary Outcomes: BMI, body weight, fat mass, waist circumference, hip circumference, and waist-to-hip ratio (WHR); Secondary Outcomes: systolic blood pressure (SBP), diastolic blood pressure (DBP), high-density lipoprotein cholesterol (HDL-C), low-density lipoprotein cholesterol (LDL-C), total cholesterol (TC), triglycerides (TG), HbA1c, fasting blood glucose (FBG), fasting insulin (FI), adiponectin (ADPN), leptin (LEP), tumour necrosis factor-alpha (TNF-α), interleukin-6 (IL-6), C-reactive protein (CRP) and (S) Study type: randomized controlled trials (RCTs) and controlled clinical trials] framework. The reference lists of included articles were searched for articles that met the inclusion criteria as well as the reference lists of all relevant systematic reviews.

### Eligibility criteria

Studies were considered eligible for inclusion if the following criteria were met: (i) participants were patients with T2DM and concurrent overweight (BMI 25–29.9 kg/m^2^) or obesity (BMI ≥ 30 kg/m^2^); (ii) no specified age limit for participants; (iii) the intervention used in the studies was AEDT; (iv) investigated at least one of the aforementioned primary outcomes in humans. Secondary outcomes also were included due to their association with cardiometabolic health; (v) articles provided full-text accessibility and were published in a refereed journal from inception up to 20 October 2023; (vi) no language restrictions; and (vii) studies were RCTs or controlled clinical trials. The following were excluded: (i) studies involving a mixed sample of individuals (e.g., apparently healthy individuals, non-diabetic people with overweight/obesity, or overweight/obese people without T2DM); (ii) articles where the effects of CART cannot be isolated because exercise training was involved as part of a multicomponent intervention (e.g., diet and exercise intervention); (iii) studies where the control group performed exercise; (iv) articles that did not assess the outcome measures of interest; (v) studies that used an acute exercise intervention (e.g., single bout or duration ≤ 2 weeks); and (vi) review articles, case reports, studies lacking a control group, and ambiguous or unclear data.

### Study selection

Four authors (S.B.A.L., W.S.W.G., M.H.A., and H.A.) employed a linear evaluation approach to assess eligibility criteria. They examined the names, abstracts, and full texts (in cases of uncertainty) and thoroughly evaluated the remaining articles based on qualifying criteria before deciding. In conflicts or uncertainties, a fifth author (M.G.) aided, applying the same method separately. Literature management software (EndNote X9, Clarivate Analytics, Philadelphia, PA, USA) was used to manage the literature search records.

### Data extraction

Two authors (S.B.A.L. and B.D.A.) independently sampled and extracted data from the relevant studies after reading the full text. The included studies generated substantial data that was retrieved and published, consisting of the first author, publication year, population, gender, sample size, exercise intervention details (frequency, intensity, time, type), study duration, and outcome measures.

### Risk of bias assessment

Two authors (S.B.A.L. and B.D.A.) independently assessed the risk of bias from individual studies according to the Cochrane Handbook for Systematic Reviews of Interventions [26]. The overall risk of bias assessment for each eligible study was judged considering the following factors: (i) random sequence generation; (ii) allocation concealment; (iii) blinding of participants and personnel; (iv) blinding of outcome assessors; (v) completeness of outcome data; (vi) selectivity of outcome reporting; and (vii) other biases as outlined in the Cochrane Handbook for Systematic Reviews of Interventions (Table S2). Eligible studies were classified into three levels of risk of bias (e.g., high, some concerns, and low) by the number of factors for which high, unclear, or low risk of bias potentially existed.

### Data analysis

All analyses were conducted using Review Manager 5.4 software (Cochrane Collaboration, https://revman.cochrane.org/info). A random-effects model was applied to present the outcomes and Cochran’s Q-test and the I^2^-test were used to assess heterogeneity [27]. We conducted a subgroup analysis when the I^2^ statistic was more than 50%, and a subgroup analysis on the reported comorbidities was performed. Mean differences (MD) or standardized mean differences (SMD) and 95% confidence intervals (CI) were applied to calculate the effect size. A two-sided *p* < 0.05 was considered to indicate statistical significance. The GRADEpro methodology (https://www.gradepro.org) was used to assess the reliability of the evidence for interpreting heterogeneity: “an I^2^ value of 0–40% may not be important”; “30–60% could indicate moderate heterogeneity”; “50–90% might suggest substantial heterogeneity”; “and 75–100% would indicate considerable heterogeneity” [28, 29]. Furthermore, Egger’s regression test was used to investigate any asymmetry in the funnel plot for detecting bias or heterogeneity for certain outcomes due to insufficient studies (< 10 studies of varying size) contributing to each outcome [26, 30], utilising the Comprehensive Meta-Analysis software version 4 (Biostat, Englewood, NJ, USA) [31].

## Results

### Literature search and selection

**Fig. 1:**
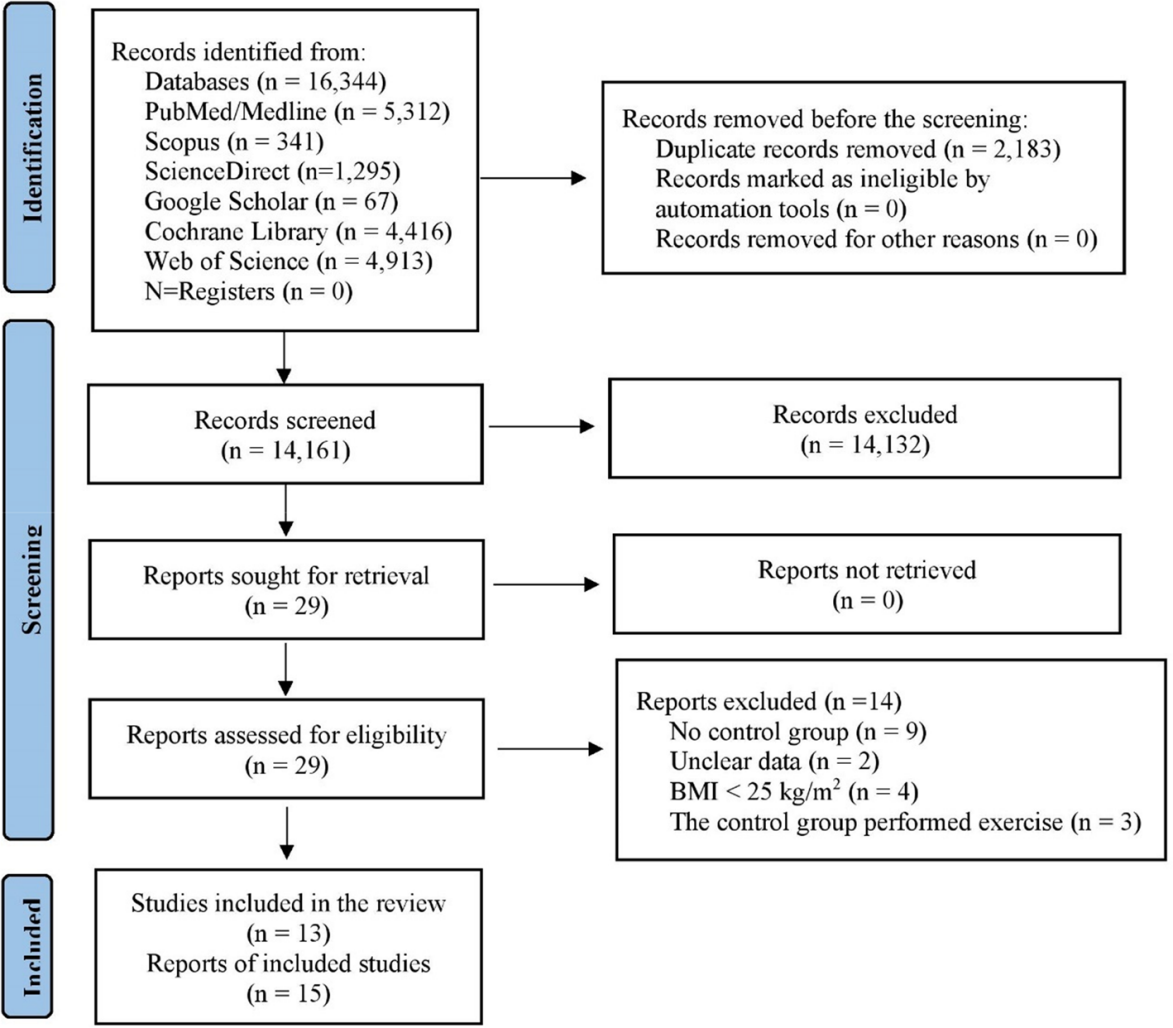
PRISMA flowchart for search strategy

From the specified databases, PubMed/Medline, Web of Science, Scopus, Science Direct, Cochrane Library, and Google Scholar (Fig. 1), a total of 16,344 studies were obtained. After removing duplicate articles, the number of studies eligible for further evaluation was reduced to 14,161. Through a review of the titles and abstracts based on predetermined inclusion and exclusion criteria, 14,132 studies were excluded. Subsequently, the full text of the remaining 29 articles was carefully examined, excluding 14 articles with reasons. Therefore, 15 records were included in this study; however, two records were subsequent studies of eligible trials included in this review. Hence, a total of 13 studies were finally included in this review, and data were extracted from 2,454 patients who met the eligibility criteria (Fig. 1 and Tables S3).

### Literature characteristics

All 13 trials were from high-income countries [32–44]. Nine out of the 13 trials recruited respondents from hospital settings [33–35, 38–40, 42–44]. In two trials, participants were recruited by direct advertising [41]. Meanwhile, in one trial, information regarding the recruitment of the participants was not provided [32, 37]. In the included trials, patients were randomly allocated to intervention and controls received ST. As for the duration of interventions, one trial lasted two years [39], six trials lasted one year [32, 33, 37, 40, 41, 44], three trials lasted 24 weeks [35, 38, 42], one trial lasted 12 weeks [43], and two trials lasted eight weeks [34, 36]. Characteristics of the included studies are shown in detail in Table S3.

### Risk of bias assessment results

**Fig. 2:**
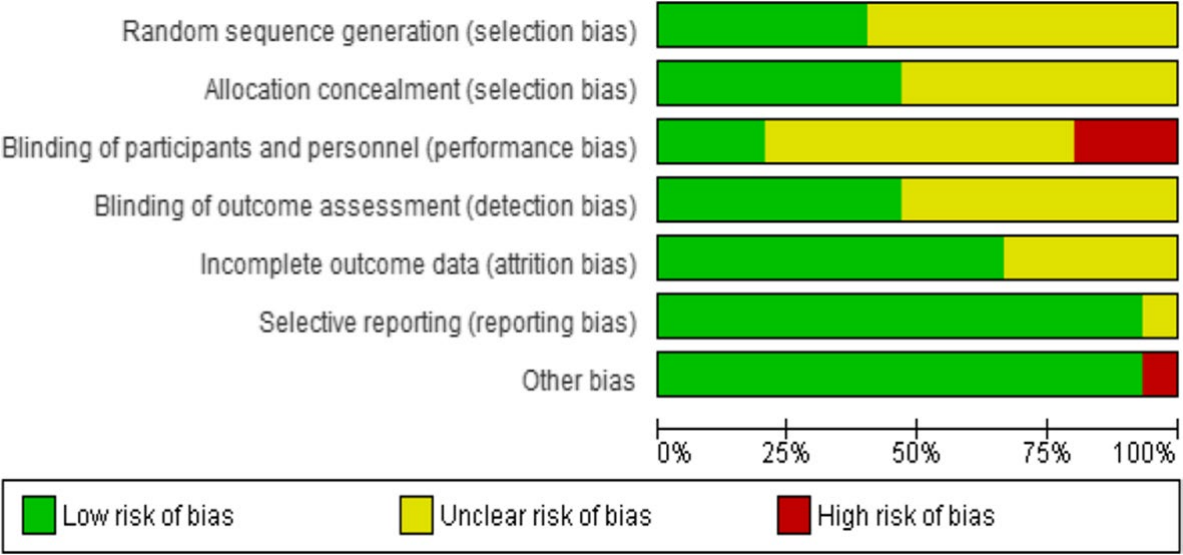
Summary of the risk of bias assessment

**Fig. 3:**
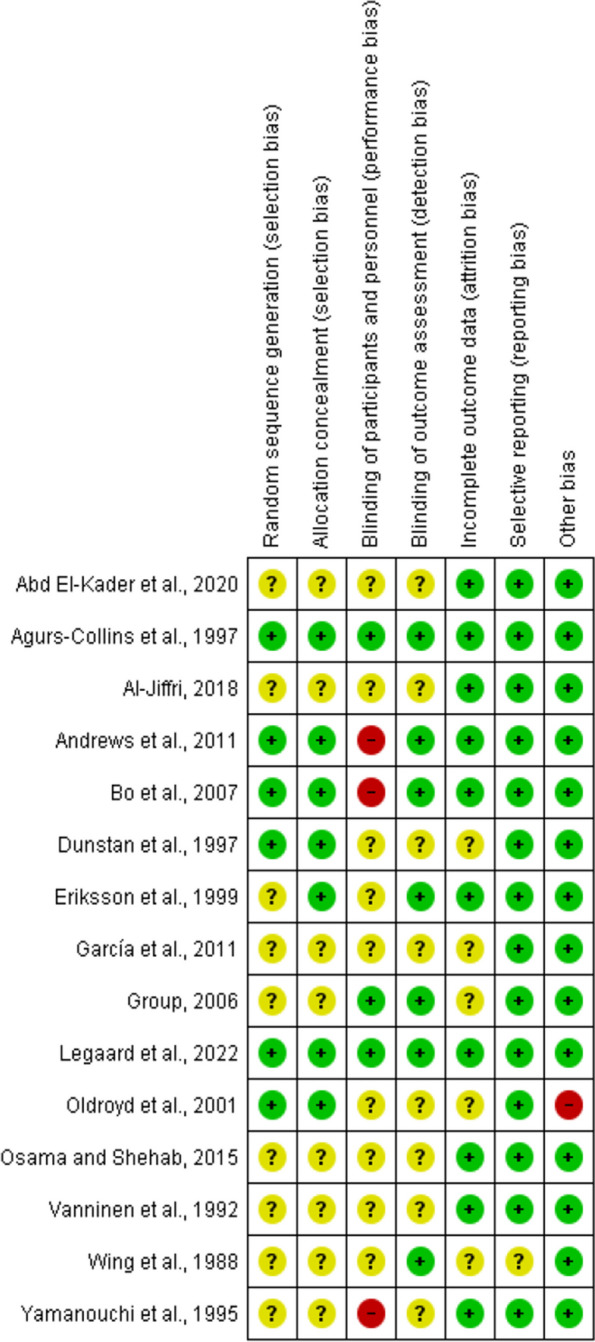
Risk of bias assessment results

The summary of the risk of bias assessment is shown in Fig. 2. Details of the risk of bias judgment per domain for each study are provided in Fig. 3. Specifically, the large majority of eligible studies demonstrated concerns about the randomization, allocation concealment, blinding of participants/personnel and outcome assessment. On the other side, the majority of studies showed a low risk of bias in missing outcome data, selective reporting processes, and other biases.

### Quality of evidence assessment

In summary, the present findings are based on low- to moderate-quality evidence according to the GRADEpro application. A summary of the evidence’s quality, the degree of the effect, and the source of information utilised in the assumed risk is shown in Table S4.

### Primary outcomes

#### Anthropometrics and body composition

**Fig. 4:**
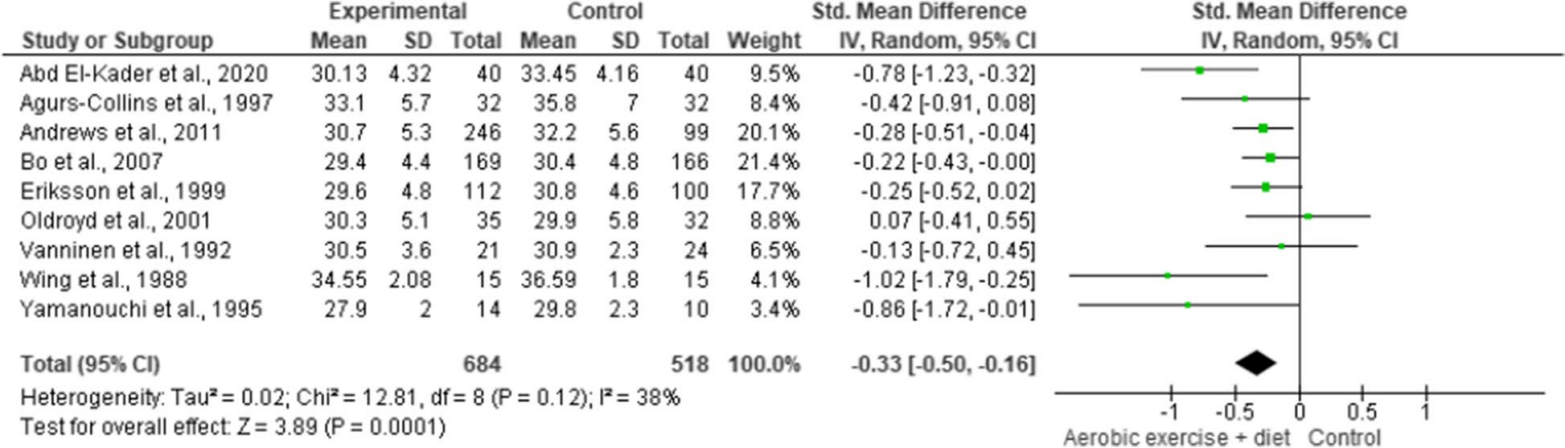
Impact of AEDT on BMI

BMI was reported in nine trials involving 1,202 participants and showing low-quality evidence. AEDT demonstrated a significant reduction in BMI compared to ST (Fig. 4 and Table S2).

Body weight was included in nine trials (*n* = 2,071) demonstrating low-quality evidence.

**Fig. 5:**
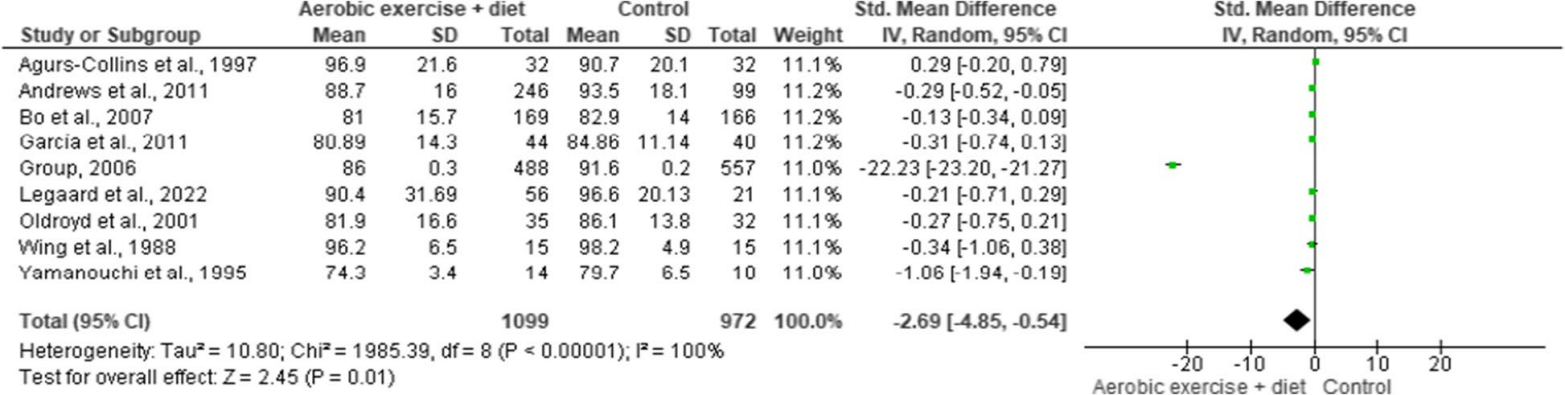
Impact of AEDT on body weight

AEDT exhibited a reduction in body weight compared to ST (Fig. 5 and Table S2).

**Fig. 6:**
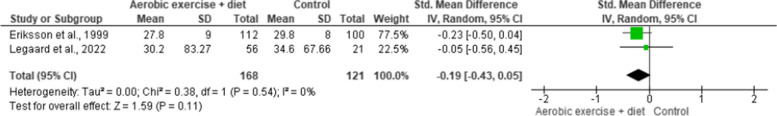
Impact of AEDT on fat mass

Fat mass was assessed in two trials recruiting 289 participants and showing moderate-quality evidence. No difference was found in fat mass between AEDT and ST (Fig. 6 and Table S2).

**Fig. 7:**
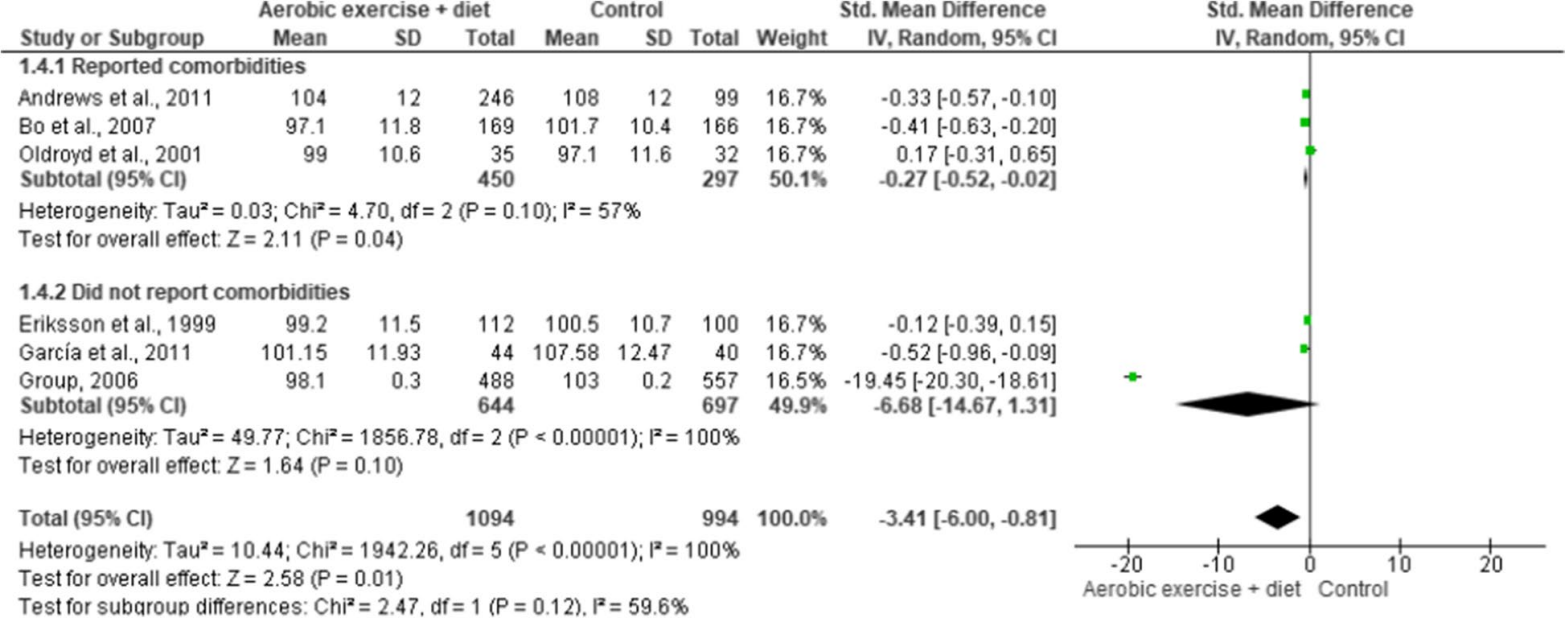
Impact of AEDT on waist circumference

**Fig. 8:**
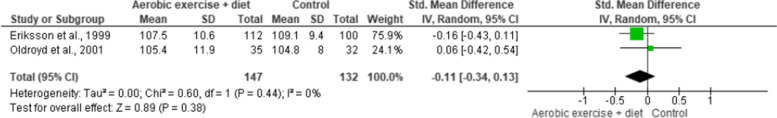
Impact of AEDT on the hip circumference

Waist and hip circumferences were measured in six (*n* = 2,088) and two (*n* = 279) trials, respectively, demonstrating low-quality evidence. AEDT showed a reduction in waist circumference and no difference was observed in hip circumference compared to ST (Figs. 7 and 8, Table S2). The subgroup analysis for waist circumference based on reported comorbidities (three trials, *n* = 747) showed that AEDT reduced waist circumference compared to ST. In trials without comorbidities, no difference was found between AEDT and ST.

**Fig. 9:**
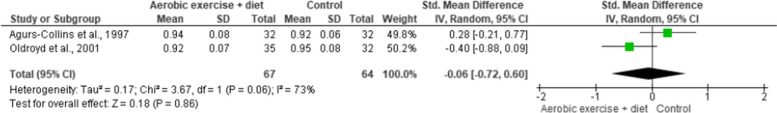
Impact of AEDT on WHR

WHR was evaluated in two trials involving 131 participants and showed low-quality evidence. No meaningful difference was detected in WHR between AEDT and ST (Fig. 9 and Table S2).

### Secondary outcomes

#### Blood pressure

**Fig. 10:**
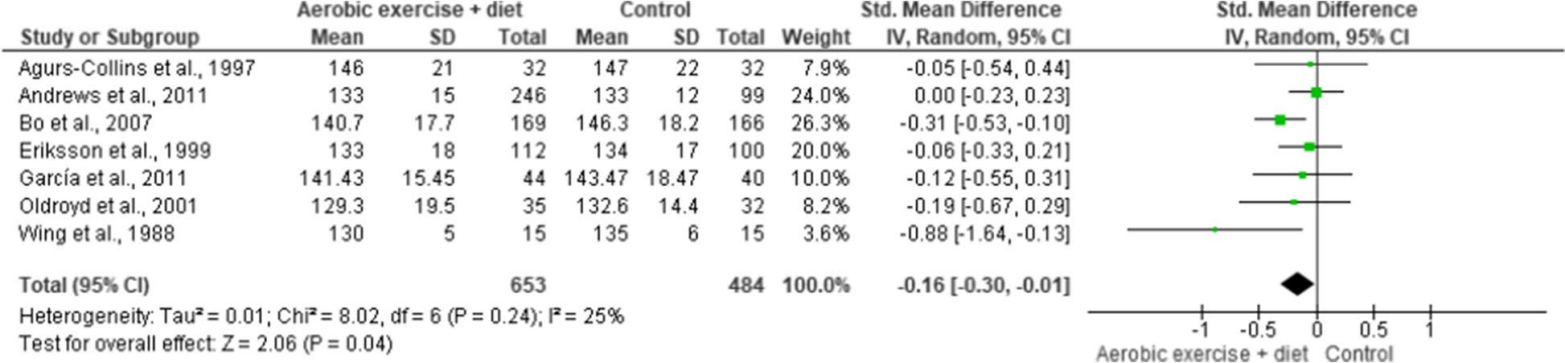
Impact of AEDT on SBP

**Fig. 11:**
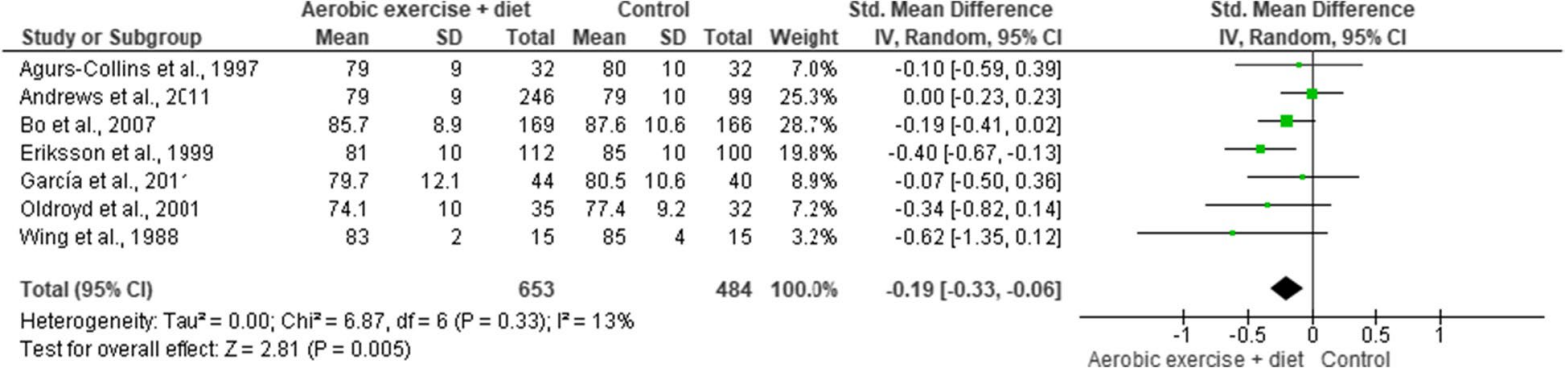
Impact of AEDT on DBP

SBP and DBP were reported in seven trials involving 1,137 participants and demonstrating moderate-quality evidence. AEDT induced more favourable reductions in SBP and DBP than ST (Figs. 10 and 11, Table S2).

#### Lipid metabolism

**Fig. 12:**
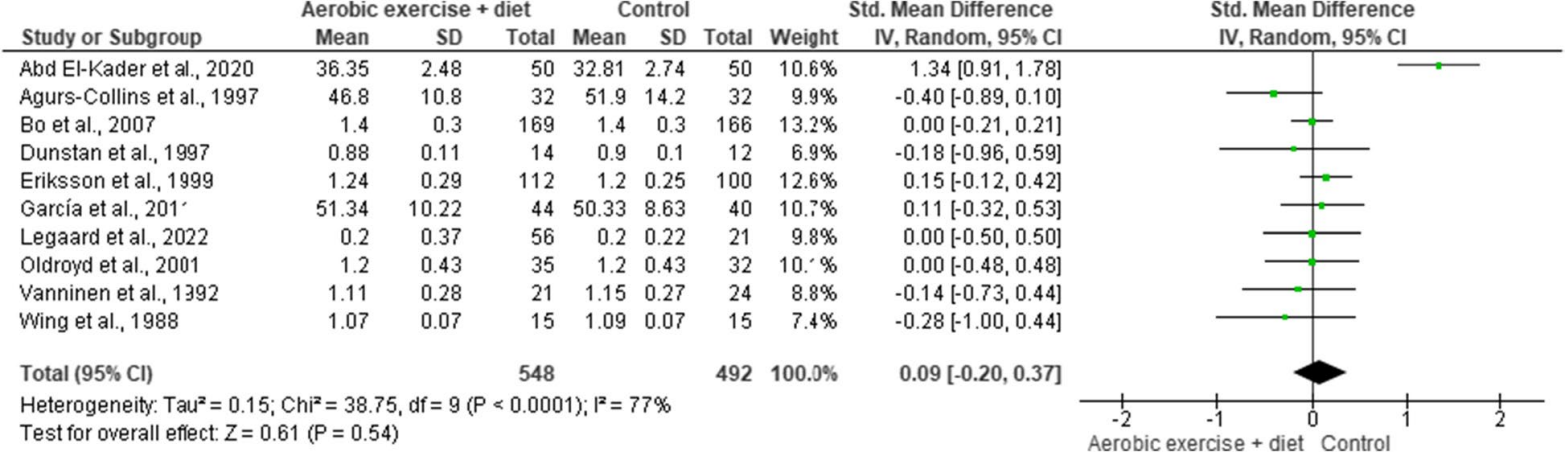
Impact of AEDT on HDL-C

**Fig. 13:**
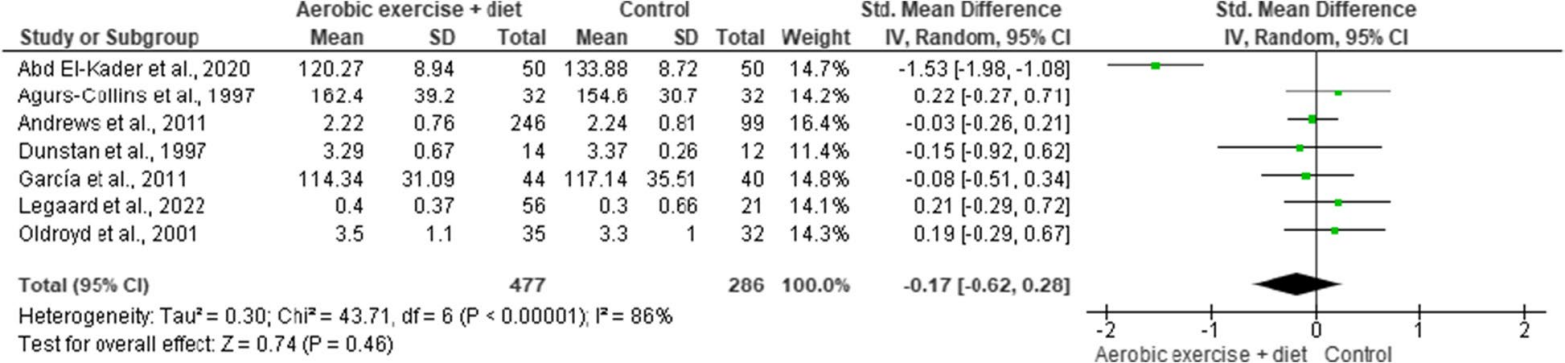
Impact of AEDT on LDL-C

HDL-C and LDL-C were included in 10 (*n* = 1,040) and seven (*n* = 763) trials, respectively, showing low-quality evidence. No substantial differences were reported in HDL-C and LDL-C between AEDT and ST (Figs. 12 and 13, Table S2).

**Fig. 14:**
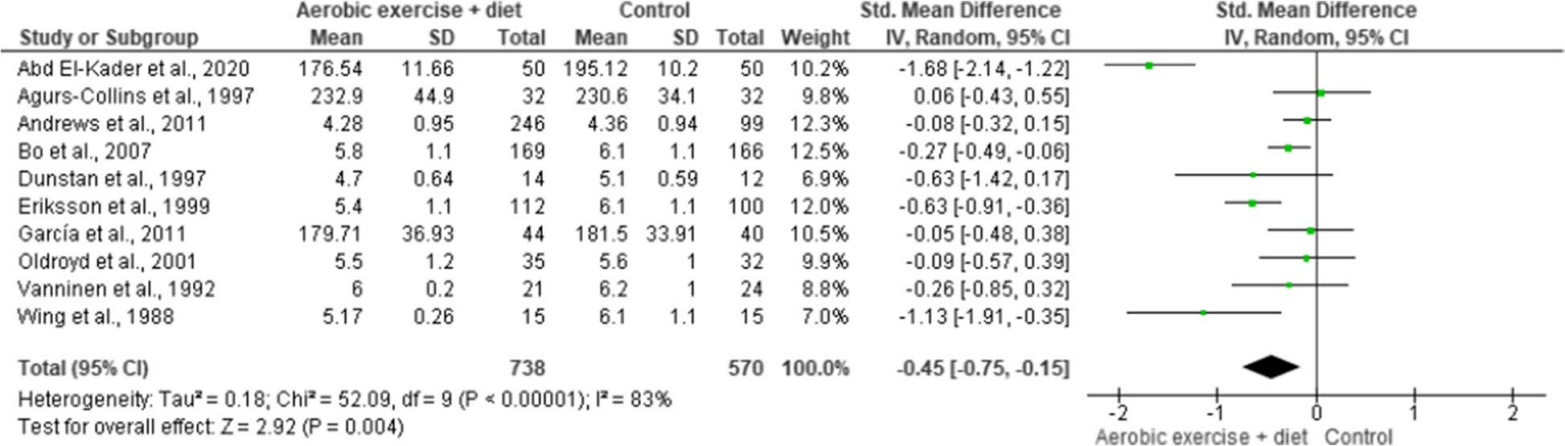
Impact of AEDT on TC

**Fig. 15:**
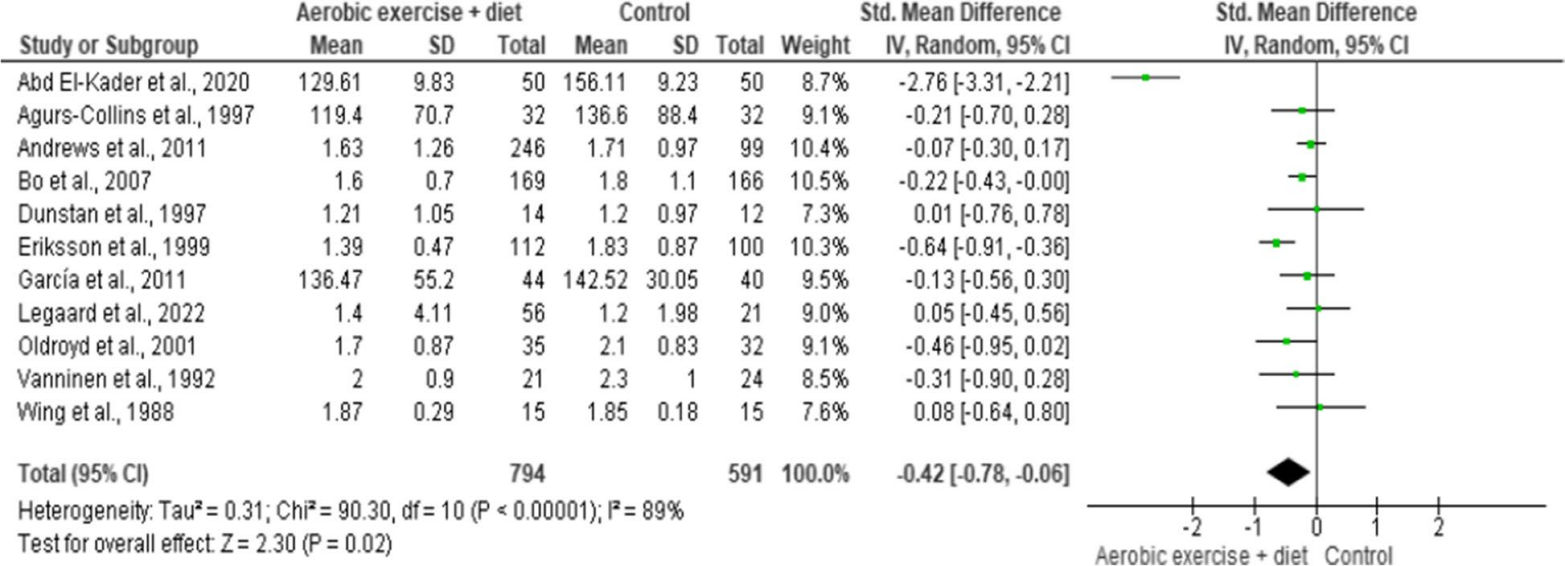
Impact of AEDT on TG

TC and TG were assessed in 10 (*n* = 1,308) and 11 (*n* = 1,385) trials demonstrating low-quality evidence. AEDT provoked more beneficial reductions in TC and TG compared to ST (Figs. 14 and 15, Table S2).

#### Glucose metabolism

**Fig. 16:**
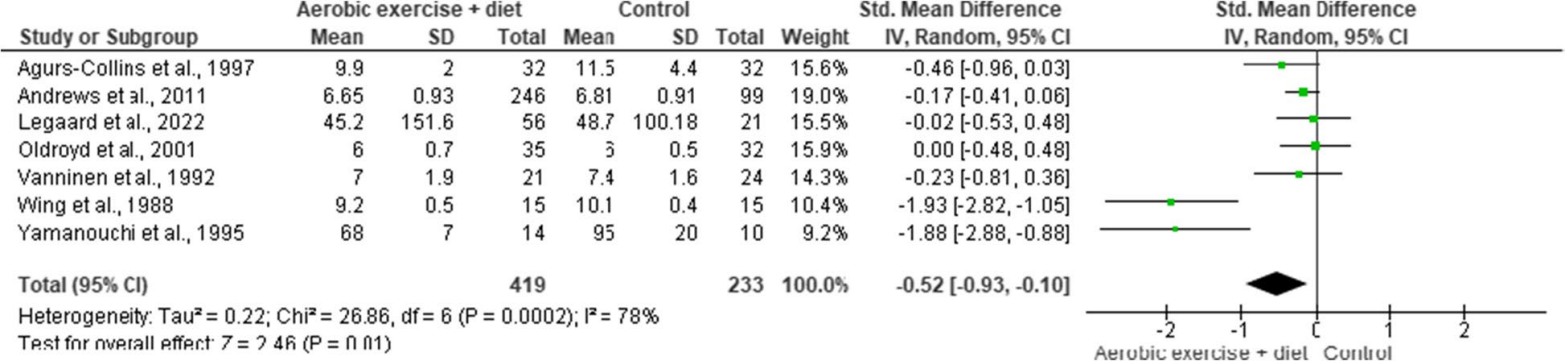
Impact of AEDT on HbA1c

**Fig. 17:**
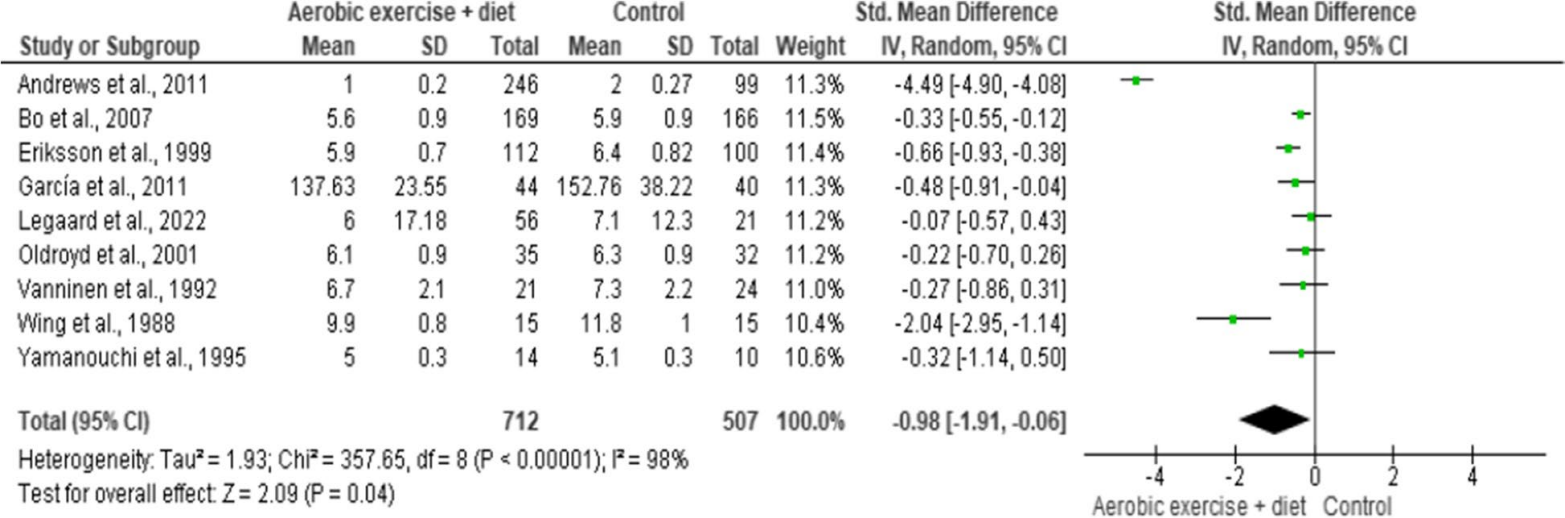
Impact of AEDT on FBG

**Fig. 18:**
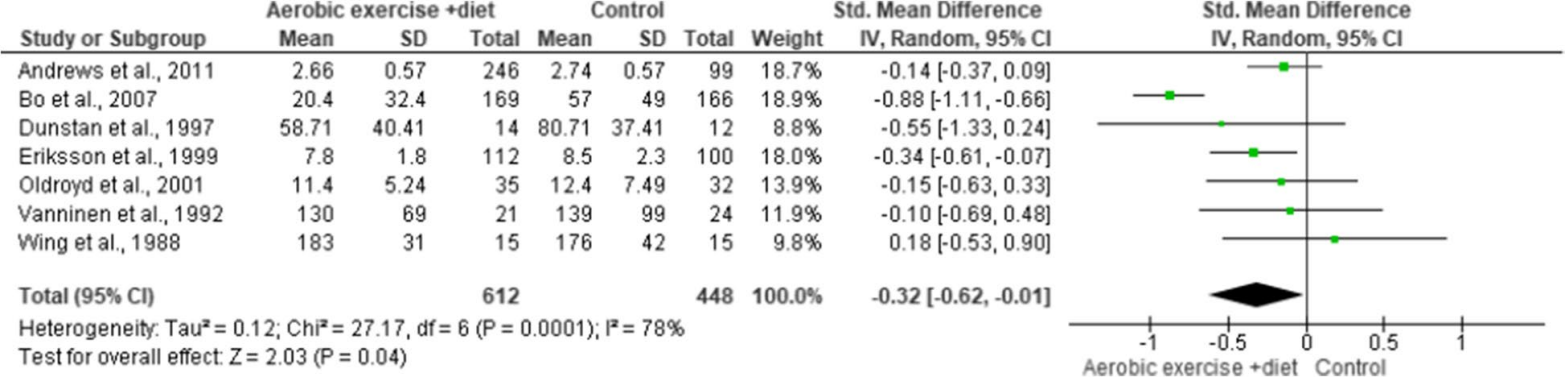
Impact of AEDT on FI

HbA1c, FBG and FI were evaluated in seven (*n* = 652), nine (*n* = 652) and seven (*n* = 1,060) trials, respectively, showing low-quality evidence. AEDT displayed beneficial alterations in HbA1c, FBG and FI compared to ST (Figs. 16, 17 and 18 and Table S2).

#### Adipose tissue dysfunction

ADPN and LEP were reported in one trial recruiting 80 participants [43]. AEDT exhibited a favourable increase in ADPN (MD 1.39, 95% CI 0.90 to 1.89; *p* < 0.001) and a favourable reduction in LEP (MD -7.31, 95% CI -9.16 to -5.46; *p* < 0.001) compared to ST, showing moderate-quality evidence (Table S2).

#### Inflammation

**Fig. 19:**
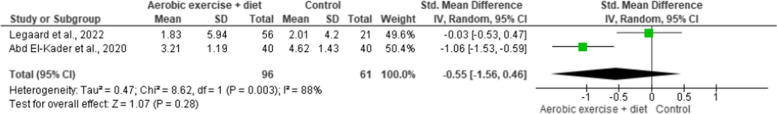
Impact of AEDT on TNF-α

TNF-α, IL-6 and CRP were reported in two (*n* = 157), one (*n* = 80) [43] and one (*n* = 80) trial [40], respectively, demonstrating low-quality evidence. No meaningful difference was found in TNF-α between AEDT and ST (Fig. 19 and Table S2), whereas AEDT exhibited significant reductions in IL-6 (MD -0.55, 95% CI -0.92 to -0.18; *p* = 0.003) and CRP (MD -0.46, 95% CI -0.68 to -0.25; *p* < 0.001) compared to ST.

### Publication bias

Although there were 15 included reports, we were not able to construct a funnel plot for detecting bias or heterogeneity for certain outcomes due to insufficient studies (< 10 studies of varying size) contributing to each outcome [26]. Hence, a funnel plot using random effects was generated to investigate potential publication bias in studies evaluating HDL-C, TG, and TC. The number of studies for each outcome varied (≥ 10 studies of varying sizes), as depicted in (Figures S1, S2, and S3) [26]. However, our analysis revealed no significant publication bias for HDL-C (*p* = 0.86), TG (*p* = 0.54), and TC (*p* = 0.40), as determined using Egger’s regression test [30].

## Discussion

In this review, we present, for the first time to the best of our knowledge, evidence about the effectiveness of AEDT on several cardiometabolic health-related indices. The present results indicate that AEDT exerts beneficial changes in body composition, lipid and glucose metabolism, blood pressure, adipose tissue dysfunction and inflammation in patients with T2DM and concurrent obesity. Given that aerobic training and/or diet alone have been reported as effective behaviour change strategies for inducing positive alterations in cardiovascular disease risk factors among people with T2DM and obesity [45–49], these findings demonstrate that AEDT may be considered the optimal lifestyle-related solution for persons with poor metabolic health due to the concurrent presence of T2DM and obesity.

### Anthropometry and body composition

This meta-analysis indicates that AEDT improves BMI, body weight and WC (only in populations with comorbidities), but not other anthropometric and body composition parameters, such as fat mass, hip circumference and WHR in persons with T2DM and obesity, underlining conflicting results in these primary outcomes that should be investigated further in the future. However, considering the key role of weight control in metabolic health-related benefits for people with T2DM and obesity, our findings seem to be important for populations characterized by metabolic health impairments. This can be explained by the fact that these cohorts are likely to show central obesity linked to visceral and ectopic fat resulting in chronic inflammation that promotes metabolic syndrome [50]. In general, the present results corroborate findings reported in other relevant reviews, indicating meaningful improvements in various anthropometric measurements [47, 51].

Interestingly, AEDT has been reported as one of the most effective non-pharmacological interventions for improving several anthropometric and body composition parameters among individuals with poor metabolic health [23, 49, 51, 52]. According to other relevant review studies, combined aerobic and resistance training (CT) evoked similar efficacy on anthropometry and body composition in individuals with T2DM and obesity [53, 54]. Importantly, aerobic exercise alone has been documented as a more effective strategy for reducing visceral adipose tissue than a hypocaloric diet alone. But diet alone appears to be more favourable for reducing body weight compared to aerobic exercise alone [55]. However, additional RCTs are necessary to determine whether AEDT can exhibit favourable alterations in visceral adiposity that are associated with an increased risk of cardiovascular disease morbidity and mortality [50].

### Blood pressure

Individuals with T2DM and obesity tend to present with elevated blood pressure levels, resulting in an increased risk of developing cardiovascular disease morbidity and mortality [56]. Normal blood pressure has been reported as a critical health marker for these particular populations according to the current international guidelines, since the combination of impaired glycemic control and raised blood pressure enhances the development of metabolic syndrome [57]. In our study, AEDT induced a meaningful reduction in SBP and DBP compared to ST. These findings are important since blood pressure improvements among people with T2DM and obesity are not well studied. In terms of the impact of various exercise modes, including aerobic training, on blood pressure demonstrates questionable changes in populations with impaired glycemic management and concurrent overweight/obesity [53, 58–60]. Additionally, diet may also be an effective lifestyle intervention for lowering blood pressure in these populations [61]. Thus, AEDT appears to be the optimal non-pharmacological approach for patients with obesity-related comorbidities seeking to achieve overall health benefits. Interestingly, aerobic training may not be the most beneficial type of physical exercise for reducing blood pressure in people with excessive weight [23]. Other more vigorous exercise modes have been documented as more favourable training options among people with metabolic health impairments [18–21, 60, 61].

### Lipid metabolism

Persons with T2DM and obesity tend to have impaired lipidemic profiles, enhancing cardiometabolic health complications [56]. In accordance with the current international guidelines, it is vital for these populations to maintain normal blood lipid levels, aiming to lower the risk of developing obesity-related illness [57]. In the present review, conflicting results were found in terms of the influence of AEDT on blood lipids, since substantial improvements were observed in TC and TG, but not HDL-C and LDL-C. In general, several types of exercise provide beneficial changes in lipid metabolism among persons with T2DM and/or obesity [23, 45, 62], which is an outcome aligned with the present results. This may be explained by the concurrent presence of obesity and T2DM, playing a key role in the simultaneous management of glycemic and lipidemic profiles due to chronic low-grade inflammation. Moreover, aerobic exercise alone does not seem as the optimal exercise strategy for inducing favorable changes in lipid homeostasis in people with excessive weight [23]. This remark partially agrees with the results reported in a relevant review, indicating beneficial alterations in LDL-C, but not HDL-C, TC and TG among patients with T2DM following aerobic exercise [45]. Also, diet plays an impactful role in lowering blood lipids in this population [63]. That being said, AEDT appears as a powerful lifestyle intervention for improving lipid metabolism indicators in patients with common metabolic health complications.

### Glucose metabolism

According to the present findings, AEDT demonstrated a significant reduction in HbA1c, FBG and FI in persons with T2DM and obesity. Noteworthy, substantial improvements in glycemic control result in a lower risk of developing diabetes-related illness. This finding is in line with results reported in previous meta-analyses examining the influence of exercise on glucose homeostasis in individuals with T2DM and/or obesity [53, 64]. Previous meta-analyses investigating the effects of aerobic exercise and/or diet on FBG and HbA1c reported meaningful reductions in T2DM patients [47, 51]. It is worth mentioning that aerobic exercise alone has not been documented as one of the most beneficial types of exercise for improving various glucose metabolism indicators in adults with obesity compared to other exercise modes [22, 23, 53]. However, AEDT appears to be the most effective strategy for increasing insulin sensitivity with or without weight loss compared to aerobic or diet alone among populations with overweight/obesity [65, 66]. Also, the metabolic benefits of regular aerobic exercise have been reported as more important than those observed for dieting regarding the impact on glycemic control linked to a pro-inflammatory profile of adipokines in persons with T2DM and/or obesity. Nevertheless, healthy dietary patterns can improve glycemic management and attenuate T2DM complications [67]. Taking these facts into account, the favourable role of AEDT in glycemia may be partly explained by the presence of visceral adiposity that is frequently observed in people with metabolic health complications [68]. Hence, such AEDT-induced adaptations may be associated with the potential activation of some key molecular mechanisms responsible for regulating glucose metabolism linked to visceral and ectopic fat [69, 70]. In summary, non-pharmacological anti-obesity agents, such as regular physical exercise, patient-centred diet and proper supplementation can be used in conjunction with prescribed medications to noticeably improve T2DM biomarkers [71, 72].

### Adipose tissue dysfunction

Adipose tissue plays an important role in lipid and glucose metabolism. Importantly, adipose tissue becomes dysfunctional in obesity, promoting a pro-inflammatory, dyslipidemic and insulin-resistant environment that enhances T2DM [73]. Also, increased circulating LEP concentrations and decreased blood levels of ADPN are common among individuals characterized by metabolic health complications [74]. In our review, AEDT exhibited significant improvements in LEP and ADPN; however, this outcome is supported by limited data since only one eligible study was included in the present meta-analysis. In general, RCTs investigating adipose tissue dysfunction in patients with T2DM and obesity are scarce. However, emerging evidence has underlined that exercise-induced adaptations of adipose tissue indicate beneficial alterations in lipolysis, glucose uptake, and mitochondrial and endocrine activity [75]. Noteworthy, AEDT can improve metabolic homeostasis and diminish the risk of developing cardiometabolic health-related complications, since adipose tissue demonstrates notable plasticity in response to lifestyle interventions integrating diet and exercise into external stimuli [76]. Nonetheless, further research in more depth is warranted, aiming to determine whether AEDT affects adipose tissue dysfunction. Such a future research strategy points to the investigation of the role of AEDT-like protocols not only in LEP and ADPN but also in other adipocytokines, such as resistin, chemerin, vaspin and omentin.

### Inflammation

People with metabolic health impairments tend to demonstrate elevated inflammatory markers since the fat cells’ hyperplasia and hypertrophy due to a progressive accumulation of triglycerides promote pro-inflammatory state which activates the production of reactive oxygen species [68]. AEDT has been broadly reported as an effective lifestyle intervention for individuals with poor metabolic health linked to chronic inflammation [69, 77–80]. In our study, we found meaningful AEDT-induced improvements in IL-6 and CRP, but not in TNF-α. Such adaptations are important since individuals with T2DM and obesity are likely to present with several cardiovascular and metabolic abnormalities linked to raised oxidative stress, impaired redox status, reduced insulin sensitivity and decreased cardiorespiratory fitness due to chronic inflammation of adipose tissue by affecting immunity [68]. Moreover, diet interventions alone can provide T2DM patients with anti-inflammatory benefits [81] but also aerobic exercise alone provokes favourable changes in inflammatory markers in individuals with T2DM and/or obesity [22, 23, 60, 82–85]. Thus, AEDT-like protocols may be an effective behaviour change strategy for lowering chronic inflammation among people with metabolic health complications. Taking these observations into consideration, our results show strong evidence considering the AEDT-induced beneficial alterations in diabetes-related inflammation, playing a key role in attenuating numerous cardiovascular disease risk factors in persons with T2DM and obesity.

### Implications for future research

Given that AEDT appears to be a beneficial lifestyle intervention for persons with T2DM and obesity with regard to advances in various cardiometabolic health-related parameters, there is a lack of robust evidence on the implementation of AEDT in the real world. Despite the latest international recommendations for persons with T2DM [67, 77, 86, 87], further study is necessary to detect the optimal exercise training parameters, such as frequency, intensity and time, aiming to aware clinicians and practitioners of the beneficial role of prescribing AEDT to individuals with T2DM and obesity [88]. Also, future research attempts in this area should emphasize the investigation of the dose-response effects of AEDT on cardiometabolic health indicators under real-world conditions as previously reported [89]. Such a future research strategy would enhance the applicability of AEDT, showing whether the most comprehensive lifestyle intervention can be used for patients with metabolic health complications in a free-living environment.

### Limitations

Our systematic review and meta-analysis have some limitations and therefore the results should be taken into consideration with caution. Eligible studies demonstrated inconsistency concerning the aerobic training parameters used during the interventions, resulting in significant heterogeneity among the included trials. The present study demonstrates that beneficial AEDT-induced adaptations are existent primarily among adults. Thus, present findings cannot be generalized to other age groups, such as children, adolescents and the elderly with T2DB and obesity. Considering the included outcome measures, the role of AEDT in a wide spectrum of overall health-related indicators of this particular population is not yet clear due to the lack of robust evidence regarding physical function and mental health.

### Conclusions

This meta-analysis provides important insights into the implementation of an adjunct, non-pharmacological treatment option that warrants appropriate attention from clinicians and practitioners within the clinical care of patients characterized by metabolic health impairments [88]. The findings show clear evidence that AEDT has a key role in improving various aspects of cardiometabolic health, such as body composition, blood pressure, lipid homeostasis, glucose homeostasis, adipose tissue dysfunction and chronic inflammation in patients with T2DM and obesity. Additional trials with robust methodological design are necessary to investigate the dose-response effects, exercise training parameters configuration and mechanisms behind these beneficial responses. The present review also indicates the need for additional RCTs to examine physical function and mental health-related outcome measures to intricate the AEDT-induced effects for individuals with T2DM and obesity.

### Supplementary Information

**Additional file 1:** **Table S1. **Search strategy. **Table S2. **Risk of bias assessment. **Table S3. **Characteristics of the included studies. **Table S4**. Summary of finding using GRADE quality assessment. **Figure S1.** Forest plot of the effects of AEDT on HDL-C among obese T2DM showing no significant publication bias (Egger’s *p* = 0.86). **Figure S2.** Forest plot of the effects of AEDT on TG among obese T2DM showing no significant publication bias (Egger’s *p* = 0.54). **Figure S3.** Forest plot of the effects of AEDT on TC among obese T2DM showing no significant publication bias (Egger’s *p* = 0.40).

## Data Availability

Data are available from the corresponding author on reasonable request.
