# Supplementary material for: Effects of combined aerobic exercise and diet on cardiometabolic health in patients with obesity and type 2 diabetes: a systematic review and meta-analysis
Source: BMC Sports Sci Med Rehabil. 2023 Dec 4;15:165. doi: 10.1186/s13102-023-00766-5 (PMC10696788; doi:10.1186/s13102-023-00766-5)
Supplement: Supplementary file 1 — Additional file 1: Table S1. Search strategy. Table S2. Risk of bias assessment. Table S3. Characteristics of the included studies. Table S4. Summary of finding using GRADE quality assessment. Figure S1. Forest plot of the effects of AEDT on HDL-C among obese T2DM showing no significant publication bias (Egger’s p = 0.86). Figure S2. Forest plot of the effects of AEDT on TG among obese T2DM showing no significant publication bias (Egger’s p = 0.54). Figure S3. Forest plot of the effects of AEDT on TC among obese T2DM showing no significant publication bias (Egger’s p = 0.40). [file 13102_2023_766_MOESM1_ESM.docx]

**Supplementary Material**

**Table S1.** Search strategy.

| **#** | **Database** | **Algorithm** |
| --- | --- | --- |
| 1 | PubMed | (((Exercise [Title/Abstract]) OR (Training [Title/Abstract])) AND (obesity [Title/Abstract])) AND (diabetes [Title/Abstract])) AND (diet [Title/Abstract]) |
| 2 | Scopus | title-ABS (exercise OR training) AND title-ABS (diabetes) AND title- ABS (obesity) AND title-ABS (diet) |
| 3 | Google Scholar | allintitle(Exercise OR Training) (obesity) (diabetes) (diet) |
| 4 | Cochrane Library | (Exercise OR Training) (diabetes) (obesity) (diet) |
| 5 | Web of Science | (Exercise) OR AB= (training) AND AB= (obesity) AND AB= (diabetes) AND AB=(diet) |
| 6 | Science Direct | (Exercise OR Training) (diabetes) (obesity) (diet) |

**Table S2.** Risk of bias assessment.

1. (Vanninen et al., 1992)

| Bias | Authors’ judgement | Support for judgement |
| --- | --- | --- |
| Random sequence generation (selection bias) | Unclear risk | Patients were randomly placed in one of two groups for the comparison of intensified treatment (the intervention group) and standard treatment (the conventional group) |
| Allocation concealment (selection bias) | Unclear risk | After a 3-month basic education programmed, 78 patients (45 men, 33 women) were randomly placed in an intervention or conventional group. |
| Blinding of participants and personnel (performance bias) All outcomes | Unclear risk | Information concerning blinding of the participants were not provided |
| Blinding of outcome assessment (detection bias) All outcomes | Unclear risk | Information concerning blinding of the assessor were not provided |
| Incomplete outcome data (attrition bias) All outcomes | Low risk | Seven patients in the intervention group did not perform the exercise tests at 0 and 12 months due to various reasons and in one patient the respiratory gas exchange measurements were unreliable, and these patients were excluded from the analyses  as well. |
| Selective reporting (reporting bias) | Low risk | Expected outcomes were reported |
| Other bias | Low risk | Other biases have not been identified |

1. (Yamanouchi et al., 1995)

| Bias | Authors’ judgement | Support for judgement |
| --- | --- | --- |
| Random sequence generation (selection bias) | Unclear risk | Patients were divided into two groups: intervention and control group. |
| Allocation concealment (selection bias) | Unclear risk | Patients were divided into two groups: intervention and control group. |
| Blinding of participants and personnel (performance bias) All outcomes | High risk | The participants were not blinding |
| Blinding of outcome assessment (detection bias) All outcomes | Unclear risk | Information concerning blinding of the assessor were not provided |
| Incomplete outcome data (attrition bias) All outcomes | Low risk | All the participant completed the study |
| Selective reporting (reporting bias) | Low risk | Expected outcomes were reported |
| Other bias | Low risk | Other biases have not been identified |

3. (Wing et al., 1988)

| Bias | Authors’ judgement | Support for judgement |
| --- | --- | --- |
| Random sequence generation (selection bias) | Unclear risk | Patients were randomly assigned to intervention and control group. |
| Allocation concealment (selection bias) | Unclear risk | No information |
| Blinding of participants and personnel (performance bias) All outcomes | Unclear risk | Information concerning blinding of participants were not provided |
| Blinding of outcome assessment (detection bias) All outcomes | Low risk | The assessor was blinding |
| Incomplete outcome data (attrition bias) All outcomes | Unclear risk | No information on reasons for  missing data provided |
| (reporting bias) | Unclear risk | Insufficient information provided. |
| Other bias | Low risk | Other biases have not been identified |

4. (Agurs-Collins et al., 1997)

| Bias | Authors’ judgement | Support for judgement |
| --- | --- | --- |
| Random sequence generation (selection bias) | Low risk | Patients were assigned randomly (1:1 ratio within  the medication strata) to either the intervention (a weight loss and exercise program) or usual care croup. |
| Allocation concealment (selection bias) | Low risk | Randomization was supervised by the study statistician, and dividing them randomly into two groups. |
| Blinding of participants and personnel (performance bias) All outcomes | Low risk | The participants were blinding |
| Blinding of outcome assessment (detection bias) All outcomes | Low risk | The assessor was blinding |
| Incomplete outcome data (attrition bias) All outcomes | Low risk | Nine patients did not complete the study due to the medical reasons, of which seven in the control and two in the intervention group. |
| Selective reporting (reporting bias) | Low risk | Expected outcomes were reported |
| Other bias | Low risk | Other biases have not been identified |

5. (Dunstan et al., 1997)

| Bias | Authors’ judgement | Support for judgement |
| --- | --- | --- |
| Random sequence generation (selection bias) | Low risk | Block randomization was used to allocate  subjects’ intervention or control groups |
| Allocation concealment (selection bias) | Low risk | Block randomization was used to allocate  subjects’ intervention or control groups |
| Blinding of participants and personnel (performance bias) All outcomes | Unclear risk | Information concerning blinding of participants were not provided |
| Blinding of outcome assessment (detection bias) All outcomes | Unclear risk | No information |
| Incomplete outcome data (attrition bias) All outcomes | Unclear risk | Six subjects withdrew due  to either changes in medication or other  commitments. However, the trial did not state to which group they are belonged |
| Selective reporting (reporting bias) | Low risk | Expected outcomes were reported |
| Other bias | Low risk | Other biases have not been identified |

6. (Legaard et al., 2022)

| Bias | Authors’ judgement | Support for judgement |
| --- | --- | --- |
| Random sequence generation (selection bias) | Low risk | Participants were randomized (2:1) stratified by sex, to the U-turn lifestyle intervention or standard care control group. The  computer-generated random number sequence used for randomization was created by an independent statistician and managed by an external individual with no study involvement. The sequence was concealed on a password-protected computer and upon request from the study nurse, the data manager would provide allocation corresponding to the participant number. |
| Allocation concealment (selection bias) | Low risk | E Participants were randomized (2:1) stratified by sex, to the U-turn lifestyle intervention or standard care control group. The  computer-generated random number sequence used for randomization was created by an independent statistician and managed by an external individual with no study involvement. The sequence was concealed on a password-protected computer and upon request from the study nurse, the data manager would provide allocation corresponding to the participant number. |
| Blinding of participants and personnel (performance bias) All outcomes | Low risk | The participants were blinding |
| Blinding of outcome assessment (detection bias) All outcomes | Low risk | The assessor was blinding |
| Incomplete outcome data (attrition bias) All outcomes | Low risk | Four patients dropped out (two in each group)  Due to loss of contact and withdrew |
| Selective reporting (reporting bias) | Low risk | Expected outcomes were reported |
| Other bias | Low risk | Other biases have not been identified |

7. (Group, 2006)

| Bias | Authors’ judgement | Support for judgement |
| --- | --- | --- |
| Random sequence generation (selection bias) | Unclear risk | Adaptative randomization is stratified by clinical center |
| Allocation concealment (selection bias) | Unclear risk | no information |
| Blinding of participants and personnel (performance bias) All outcomes | Low risk | Investigators and participants remain masked to primary outcome data until progression to diabetes is confirmed.” “Assignments to metformin and placebo were double-blinded” |
| Blinding of outcome assessment (detection bias) All outcomes | Low risk | Investigators and participants remain masked to primary outcome data until progression to diabetes is confirmed.” “Assignments to metformin and placebo  were double-blinded” |
| Incomplete outcome data (attrition bias) All outcomes | Unclear risk | No information provided |
| Selective reporting (reporting bias) | Low risk | Expected outcomes were reported |
| Other bias | Low risk | Other biases have not been identified |

8. (Bo et al., 2007)

| Bias | Authors’ judgement | Support for judgement |
| --- | --- | --- |
| Random sequence generation (selection bias) | Low risk | Participants were stratified according to age, sex, education level. The randomization procedure was automatically performed by a statistician using a SAS programme developed to minimize the differences between the two groups for all stratifying variables.” |
| Allocation concealment (selection bias) | Low risk | Random allocation with a minimization algorithm was centrally performed in a single step. The researchers then received the two lists of nominative data. |
| Blinding of participants and personnel (performance bias) All outcomes | High risk | Because of the nature of the intervention, blinding participants  was not possible. |
| Blinding of outcome assessment (detection bias) All outcomes | Low risk | family physicians, the physicians who collected data, the dietician, and the laboratory personnel were blinded to the group assignment. |
| Incomplete outcome data (attrition bias) All outcomes | Low risk | written informed consent to participate was not given by 18 of 187 (9.6%) and 22 of 188 (11.7%) subjects from the intervention group and the control group, respectively. |
| Selective reporting (reporting bias) | Low risk | Expected outcomes were reported |
| Other bias | Low risk | Other biases have not been identified |

9. (Eriksson et al., 1999)

| Bias | Authors’ judgement | Support for judgement |
| --- | --- | --- |
| Random sequence generation (selection bias) | Unclear risk | The randomization was stratified by center, sex and the mean 2-h plasma glucose concentration (7.8±9.4 mmol/l or 9.5±11.0 mmol/l). |
| Allocation concealment (selection bias) | Low risk | randomly assigned to the intervention group or the control group by the study physician, with the using computer of a randomization list” |
| Blinding of participants and personnel (performance bias) All outcomes | Unclear risk | Information concerning blinding of participants were not provided |
| Blinding of outcome assessment (detection bias) All outcomes | Low risk | The nurses who scheduled the study visits did not have access to the randomization list... Laboratory staff did not know the subjects’ group assignments, and the subjects were not informed of their plasma glucose concentrations during follow-up unless diabetes was diagnosed |
| Incomplete outcome data (attrition bias) All outcomes | Low risk | 40 subjects (8 percent) withdrew - 23 in the intervention group and 17 in the control group... 9 could not be contacted, 3 withdrew due to severe illness, 1 died, and 27 withdrew for personal reasons.” |
| Selective reporting (reporting bias) | Low risk | Expected outcomes were reported |
| Other bias | Low risk | Other biases have not been identified |

10. (Oldroyd et al., 2001)

| Bias | Authors’ judgement | Support for judgement |
| --- | --- | --- |
| Random sequence generation (selection bias) | Low risk | Eligible participants who agreed to take part were randomly allocated using a random number table to the intervention or control group at the first baseline appointment |
| Allocation concealment (selection bias) | Low risk | Researchers performing the randomization were blinded to the group allocation. |
| Blinding of participants and personnel (performance bias) All outcomes | Unclear risk | Information concerning blinding of participants were not provided |
| Blinding of outcome assessment (detection bias) All outcomes | Unclear risk | Information concerning blinding of the assessor was not provided |
| Incomplete outcome data (attrition bias) All outcomes | Unclear risk | Comment: there was a high attrition rate, not balanced between treatment groups.  Attrition rate was 23% in the treatment group and 38% in the control group after 24 months of follow-up. |
| Selective reporting (reporting bias) | Low risk | Expected outcomes were reported |
| Other bias | High risk | “A significantly larger proportion of control participants reported engaging in regular physical activity sufficient to get their heart thumping at least once a week compared with intervention participant (53% versus 24%)”  “There were fewer women (10/32 (32%) than men [22/32 (69%)] in the control group compared with the intervention  group...” |

11. (Andrews et al., 2011)

| Bias | Authors’ judgement | Support for judgement |
| --- | --- | --- |
| Random sequence generation (selection bias) | Low risk | Randomization was done according to computer-generated allocation |
| Allocation concealment (selection bias) | Low risk | Patients were assigned, in a  2:5:5 ratio, to usual care, an intensive diet intervention,  or the intensive diet intervention plus activity. |
| Blinding of participants and personnel (performance bias) All outcomes | High risk | Participants were not blinding |
| Blinding of outcome assessment (detection bias) All outcomes | Low risk | The assessor was blinding |
| Incomplete outcome data (attrition bias) All outcomes | Low risk | Six patients in each group did not complete the post treatment assessment due to loss to follow -up and family reason. |
| Selective reporting (reporting bias) | Low risk | Expected outcomes were reported |
| Other bias | Low risk | Other biases have not been identified |

12. (Osama and Shehab, 2015)

| Bias | Authors’ judgement | Support for judgement |
| --- | --- | --- |
| Random sequence generation (selection bias) | Unclear risk | Patients were randomly assigned into intervention and control group |
| Allocation concealment (selection bias) | Unclear risk | No information |
| Blinding of participants and personnel (performance bias) All outcomes | Unclear risk | No information |
| Blinding of outcome assessment (detection bias) All outcomes | Unclear risk | No information |
| Incomplete outcome data (attrition bias) All outcomes | Low risk | All the participant completed the study |
| Selective reporting (reporting bias) | Low risk | Expected outcomes were reported |
| Other bias | Low risk | Other biases have not been identified |

13. (Abd El-Kader and Al-Jiffri, 2018)

| Bias | Authors’ judgement | Support for judgement |
| --- | --- | --- |
| Random sequence generation (selection bias) | Unclear risk | Patients were randomly assigned into intervention and control group |
| Allocation concealment (selection bias) | Unclear risk | No information |
| Blinding of participants and personnel (performance bias) All outcomes | Unclear risk | No information |
| Blinding of outcome assessment (detection bias) All outcomes | Unclear risk | No information |
| Incomplete outcome data (attrition bias) All outcomes | Low risk | Three patients in the intervention group and two in the control group dropped out due to lost to follow up and family reason |
| Selective reporting (reporting bias) | Low risk | Expected outcomes were reported |
| Other bias | Low risk | Other biases have not been identified |

14. (Abd El-Kader et al., 2020)

| Bias | Authors’ judgement | Support for judgement |
| --- | --- | --- |
| Random sequence generation (selection bias) | Unclear risk | Patients were randomly assigned into intervention and control group |
| Allocation concealment (selection bias) | Unclear risk | No information |
| Blinding of participants and personnel (performance bias) All outcomes | Unclear risk | No information |
| Blinding of outcome assessment (detection bias) All outcomes | Unclear risk | No information |
| Incomplete outcome data (attrition bias) All outcomes | low | Three patients in the intervention group and four patients in the control group dropped out due to lost to follow up and family reason |
| Selective reporting (reporting bias) | Low risk | Expected outcomes were reported |
| Other bias | Low risk | Other biases have not been identified |

15. (Ferrer-García et al., 2011)

| Bias | Authors’ judgement | Support for judgement |
| --- | --- | --- |
| Random sequence generation (selection bias) | Unclear risk | Eighty-four Spanish patients aged over 60 years were randomized to participate in a home-based, combined physical exercise program or standard treatment |
| Allocation concealment (selection bias) | Unclear risk | Patients enrolled into the intervention cohort followed the standard treatment for diabetes and, a specific 24-week physical activity program. Consecutive probability sampling was performed, followed by the randomization of each patient into one of the two cohorts. Eighty-four participants were selected and distributed into 2 groups |
| Blinding of participants and personnel (performance bias) All outcomes | Unclear risk | Information concerning blinding of participants were not provided |
| Blinding of outcome assessment (detection bias) All outcomes | Unclear risk | Information concerning blinding of the assessor was not provided |
| Incomplete outcome data (attrition bias) All outcomes | Unclear risk | Ten patients withdrew during the study, four from the intervention group and six from the control group.  The trials stated that most patients who dropped out from the program did so for family reasons or due to difficulties in attending program learning sessions, however the trial did not state all the reasons for patients dropped out. Hence, intention-to-treat analysis was applied. |
| Selective reporting (reporting bias) | Low risk | Expected outcomes were reported |
| Other bias | Low risk | Other biases have not been identified |

**Table S3.** Characteristics of the included studies.

|  | **Participants’ Age (yrs), BMI (kg/m^2^) & Country** | **Reported comorbidities** | **Study Design** | **Recruitment & Grouping** | **Control Intervention** | **Test-Group Intervention & Context** | **Intervention** | **Outcome Measures** | **Pro-instrument measure** |
| --- | --- | --- | --- | --- | --- | --- | --- | --- | --- |
| 1 [27] | 53 ± 7  32 ± 4  Finland | -Hypertension  -Ischaemic heart disease  3.6% were smokers | RCT | Hospital Recruitment  N=45:  CO= 24  Ex= 21  The intervention group was treated at the outpatient clinic of the Department of Medicine, Kuopio University  Hospital. | No-exercise and maintained their normal  daily activities | Frequency: 3d/wk of AE  Duration: 30 to 50 min. Intensity: HR [11-140] bpm  Diet education, energy restriction, restriction of the intake of total fat, and especially saturated fatty acids and dietary cholesterol were implemented | 1 year | 1. Blood glucose  2. HbAlc  3. Lipid profile  4. BMI.  5. VO_2_MAX | 1. (Glucose  Auto & Stat HGA-1120 analyzer, Daiichi Co, Kyoto, Japan). (METs) calculation  2. Commercial fast protein liquid chromatography (Pharmacia Fine Chemicals AB, Uppsala, Sweden)  3. (Monotest and Test-Combination, respectively, Boehringer  Mannheim, Mannheim, FRG).  4. weight/height squared (kg. m-2)  5- |
| 2 [28] | 41.6 ± 3.5  31.2 ± 1.2  Japan | - | Clinical trial | Hospital Recruitment  N= 24  CO= 10  EX = 14 | No-exercise and maintained their normal  daily activities | AE includes walking at least 10,000 steps/day on a flat field as monitored by a pedometer (19,200 ± 2,100 steps/day).  Plus 1,000-1,600 kcal/day (54-58%carbohydrates, 17-20% protein, and 25- 26% fat). | 8 weeks | 1. Blood glucose  2. Plasm insulin  3. HbA1c  4. Body mas  5. Body weight | 1. –  2. –  3. –  4.-  5. - |
| 3 [26]  The assessor were blinding | 56.1 ± 6.4  37.5 ± 1.9  USA | - | RCT | The Recruitment  the method was not stated  N= 30  CO= 15  EX = 15 | Subjects in the diet  only condition were instructed not to change their baseline level of activity | The diet plus exercise group walked a 3-mile route for 3/week and were instructed to  exercise additionally once/week on their own. All subjects were given a daily calorie goal designed to produce  approximately 1 kg/week of weight loss. The calorie goal was calculated by taking the patient's pre-treatment weight (in kg), multiplying by 26  and subtracting 1000 calories. | 1 year | 1. Weight  2. BMI  3. Blood pressure  4. lipid profile  5. Plasma glucose and insulin  6. HbA1c | 1. Balance beam scale  2. (kg/m^2^).  3. -  4. Using enzymatic  Procedures  5. by radioimmunoassay (Ross Laboratory, Columbus, Ohio, USA)  6. (Isolab minicolumns, Akron, Ohio, USA) |
| 4 [29] | 62.4 ± 5.9  33.9 ±5.1  USA | - | RCT | Participants were recruited  through general medicine clinics and diabetes clinics, from public health department  geriatric clinics  N= 64  Ex= 32  CO= 32 | Usual care | The intervention objectives were to promote adherence to a diet with ~55-60% kcal from carbohydrate, 12-20% from protein, and <30% from fat (9), a weight loss of at least 10 lb (4.5 kg) at the rate of <2 lb  (0.9 kg) per week, and moderate AE at least 3 days per week | 6 months | 1. HbA1c  2. lipid profile  3. Blood pressure  4. BMI  5. Waist circumference  6. Hip circumference | 1. Using the BioRad  Diamant System  2. enzymatic methods  (35-37) with an Olympus AU 5000 analyze  3. Sphygmomanometer  4. By dividing  weight (in kilograms) by height (in meters)  squared  5. Was measured at the narrowest part of the trunk, if evident, or midway between the lowest rib  and the iliac crest  6. Was measured at the maximum protrusion of the buttocks. |
| 5 [30] | 52.6 ± 7.2  29.9 ± 3.0  Australia | - | RCT | Hospital outpatients  N= 26  Ex= 14  CO= 12 | Usual care | AE performed on 3  nonconsecutive days of the week in a supervised laboratory setting using cycling ergometer at 55-65% of the baseline Vo2max, plus fish and diets supplying 30% or less of total energy intake from fat  (<10% saturated fat), with the remainder distributed between carbohydrates and protein. Also, patients were advised to reduce their sodium intake to < 100 mmol/day. | 8 weeks | 1. Blood sugar  2. Weight  3. Lipid profile  4. HbA1c  5. Fasting serum insulin | 1. Using the trapezoidal method with fasting concentrations  (Incremental area) and zero as the baseline  2. Calibrated beam balance scale  3. Were determined enzymatically  4. high-performance liquid chromatography  5. EIA (enzyme immunoassay) |
| 6 [37] | 53.3 ± 9.4  32.8 ± 4.1  Denmark | Reported comorbidities | RCT | Hospital outpatients  N=77:  CO=21  Ex= 56 | Standard care | Five to six AEsessions per week, and an adjunct dietary  intervention aiming at a reduction of ~500 kcal/day (months 0–4). The diet was isocaloric from months 5–12 | 12 months | 1. Body composition  2. Blood samples  3. VO2MAX | 1. A dual-energy x-ray absorptiometry scan (Lunar Prodigy Advance,  GE Medical Systems Lunar, USA) with software (Prodigy, enCORE 2004,  version 8.8, GE Lunar Corp, USA)  2. (Mesoscale, V-Plex human proinflammatory  panel I and V-Plex human IL-1ra kit, Meso Scale Discovery, USA)  3. Monarch LC4  Bicycle (Monark Exercise, Sweden) |
| 7 [33] | 50.6 ± 10.7  34.0 ± 6.7  USA | - | RCT | Hospital outpatients  N=1045:  CO= 557  Ex= 488 | annual 30 min  individual session on healthy lifestyles | Moderate-intensity AE for  150 min a week; supervised group exercise sessions twice a week were offered.  Dietary intervention: goal 7% weight loss through a healthy low-calorie, low-fat diet. | Follow-up: average 2.8 years | 1. Weight  2. Waist circumference | 1-  2- |
| 8 [34] | 55.7 ± 5.7  29.7±4.1  Italy | Reported comorbidity | RCT | Hospital Recruitment  N=335:  CO=166  Ex=169 | family physician advice emphasizing the importance of a healthy lifestyle according to their usual clinical practice | Family physician advice plus detailed verbal and written recommendation including  individually prescribed diet and advice on exercise mainly by suggesting moderate-intensity AE, such as brisk walks for at least 150 minutes/week. Sessions had a flexible  structure, sensitive to cultural differences and patient expectations | 1 year | 1. Blood pressure  2. Weight  3. BMI  4. Waist circumference  5. lipid profile  6. Blood glucose | 1. A standard mercury sphygmomanometer  2. –  3. kg/m^2^  ^4. A plastic tape meter at the level of the umbilicus^  5. –  6 S |
| 9 [31] | Finland. |  | RCT | The study subjects were recruited through various methods, e. g. from epidemiological surveys and by opportunistic  population screenings with special emphasis on the high-risk  groups such as obese subjects and first-degree relatives of  Type II diabetic patients. Subjects were also recruited through  advertising in local newspapers  N=27:  CO=100  Ex=112 | General written and oral information to prevent DM and dietary advice | The AE included (walking, jogging, swimming,  aerobic ball games, and skiing) twice a week. Plus Low fat, high-fiber diet; goal BMI <25 or 5-10kg weight loss; <50%  carbohydrate, <30% fat, <300 mg/day cholesterol. | 1 year | 1. Waist and hip circumference  2. Blood pressure  3. Blood parameter | 1. Measured midway between the lowest rib and iliac crest and hip circumference over the great trochanters, with 0.5 cm precision with the subject in a standing position  2. A standard sphygmomanometer  3. Done in the central laboratory of the Department of  Biochemistry, National Public Health Institute, Helsinki |
| 10 [32] | 41–75  30.4 ± 5.6  UK | - |  | Hospital settings  N=65:  CO=32  Ex=35 | No intervention | Physical activity intervention: graded plan, tailored to the participants lifestyle and  designed to enable them to achieve 20-30 min of AE al least once a week. Plus Diet intervention: Reduce BMI to <25 in overweight; <=30 % of energy from fat; polyunsaturated to saturated fat ratio >=1.0; 50% from carbohydrate; >=20g per 4.2MJ dietary fiber intake | 6 months | 1. Weight  2. BMI  3. Waist and hip circumferences  4. Waist: hip ratio (WHR)  5. Blood pressure  6. Serum insulin  7. Lipid profile | 1. Measured to the nearest 0.1 kg with  the participants lightly clothed on SECA scales  (Alpha Model 770 digital, SECA, Birmingham,  UK)  2. kg/m2  3. Measured to the nearest cm with the  participants standing, using a spring-loaded tape measure  4. Calculated from the mean of two waist and  two hip measurements. 5. Mercury sphygmomanometer  6. enzyme linked immunosorbent assay (ELISA)  (DAKO Diagnostics, Ely, UK).  7. Blood samples on  a DAX analyser (Bayer, Basingstoke, UK) |
| 11 [35] | 60·1 ± 10·2  31·6 ± 5·6  UK | Reported comorbidities | RCT | By searching the records databases of  217 general practices in southwest England, and of  community-based education programmes, and by direct advertising.  N=345:  CO=99  Ex=246 | Usual care | The intensive diet intervention was aimed at enabling  patients to lose 5–10% of their initial bodyweight with encouragement to choose foods in the lower  ranges of energy density, fat content, and glycaemic  index. Plus 30 min brisk walking on at least  5 days per week. | 1 year | 1. Insulin resistance  2. Body composition  3. lipid profile | 1. Measured by homoeostasis model  Assessment  2. Were measured according to standard  Procedures.  3. Were measured according to standard  Procedures |
| 12 [23] | 36.4 ± 5.1  33.5 ± 4.2  Saudi Arabia | - | Clinical trial | Hospital Recruitment  N=100:  CO=50  Ex=50 | No exercise or diet | forty minutes moderate intensity AE sessions  on a treadmill. First 2 weeks = 60–70% of HRmax, 3rd to 12th weeks = 70–80% of HRmax. Each session was continued for  30 minutes; 3 sessions.  Plus weight loss programme about1200 kcal/day, based on a macronutrient content  <30% fat and 15% protein. | 3 months | 1. Lipid profiles | 1. Was measured on a calibrated balance scale to the nearest 0.1 kg  (HC4211, Cas Korea, South Korea)  2. Body weight /  (Height)2  3. Hitachi 7170 Autoanalyser (Tokyo, Japan) or with commercial kits (Randox). |
| 13 [24] | 50.1 ± 5.9  33.1 ± 3.2 |  | Clinical trial | Hospital settings  N=80:  CO=40  Ex=40 | - | - | - | 2. Leptin  3. Adiponectin |  |
| 14 [25] |  |  | Clinical trial | Hospital settings  N=80:  CO=40  Ex=40 | - | - | - | 4. BMI  5. IL-6  6. TNF |  |
| 15 [36] | 66.7 ± 8.0  31.3 ± 6.2  Spain | _ | RCT  Spa in | By clinical interview  N=84:  CO=40:  Ex=44 | No exercise | 45 min of moderate physical (particularly aerobic) for 5 days. Home-based physical education program plus diet comprising between 1500 and 2000 kcal/day, distributed over five meals, with 15% protein, 55% carbs, and 30% fat (less than 10% of which is saturated fat). | 24 weeks | 1. Lipid profile  2. weight | 1.  2. In (kg) |

AE; aerobic exercise; BMI; body mass index; min; minute; HRmax; maximum heart rate; RCT; randomized control trial; EX; exercise; CO; control.

**Table S4**. Summary of finding using GRADE quality assessment.

| **Outcome** | **Certainty assessment** | | | | | | | **№ of patients** | | **Effect** | **Certainty** |
| --- | --- | --- | --- | --- | --- | --- | --- | --- | --- | --- | --- |
|  | **№ of studies** | **Study design** | **Risk of bias** | **Inconsistency** | **Indirectness** | **Imprecision** | **Other considerations** | **Intervention group** | **Control group** | **Absolute (95% CI)** |  |
| BMI | 9 | RCT | serious^a^ | not serious | not serious | serious^b^ | none | 684 | 518 | SMD **0.33 lower** (0.5 lower to 0.16 lower) | ⨁⨁◯◯ Low |
| Fat mass | 2 | RCT | not serious | not serious | not serious | serious^b^ | none | 168 | 121 | SMD **0.19 lower** (0.43 lower to 0.05 higher) | ⨁⨁⨁◯ Moderate |
| Body weight | 9 | RCT | not serious | serious^c^ | not serious | serious^b^ | none | 1099 | 972 | SMD **2.69 lower** (4.85 lower to 0.54 lower) | ⨁⨁◯◯ Low |
| Waist circumference | 6 | RCT | not serious | serious^c^ | not serious | serious^b^ | none | 1094 | 994 | SMD **3.41 lower** (6 lower to 0.81 lower) | ⨁⨁◯◯ Low |
| Waist circumference reported comorbidities | 3 | RCT | serious^a^ | serious^d^ | not serious | serious^b^ | none | 450 | 297 | SMD **0.27 lower** (0.52 lower to 0.02 lower) | ⨁◯◯◯ Very low |
| Waist circumference did not report comorbidities | 3 | RCT | not serious | serious^c^ | not serious | not serious | none | 644 | 697 | SMD **6.68 lower** (14.67 lower to 1.31 higher) | ⨁⨁⨁◯ Moderate |
| Hip circumference | 2 | RCT | serious^e^ | not serious | not serious | serious^b^ | none | 147 | 132 | SMD **0.11 lower** (0.34 lower to 0.13 higher) | ⨁⨁◯◯ Low |
| Waist-to-hip ratio | 2 | RCT | serious^e^ | serious^f^ | not serious | serious^b^ | none | 67 | 64 | SMD **0.06 lower** (0.72 lower to 0.6 higher) | ⨁◯◯◯ Very low |
| SBB | 7 | RCT | not serious | not serious | not serious | serious^b^ | none | 653 | 484 | SMD **0.16 lower** (0.3 lower to 0.01 lower) | ⨁⨁⨁◯ Moderate |
| DBP | 7 | RCT | not serious | not serious | not serious | serious^b^ | none | 653 | 483 | SMD **0.19 lower** (0.33 lower to 0.06 lower) | ⨁⨁⨁◯ Moderate |
| HDL-C | 10 | RCT | not serious | serious^f^ | not serious | serious^b^ | none | 548 | 492 | SMD **0.09 higher** (0.2 lower to 0.37 higher) | ⨁⨁◯◯ Low |
| LDL-C | 7 | RCT | not serious | serious^f^ | not serious | serious^b^ | none | 477 | 286 | SMD **0.17 lower** (0.62 lower to 0.28 higher) | ⨁⨁◯◯ Low |
| Total cholesterol | 10 | RCT | not serious | serious^f^ | not serious | serious^b^ | none | 738 | 570 | SMD **0.45 lower** (0.75 lower to 0.15 lower) | ⨁⨁◯◯ Low |
| Triglyceride | 11 | RCT | not serious | serious^f^ | not serious | serious^b^ | none | 794 | 591 | SMD **0.42 lower** (0.78 lower to 0.06 lower) | ⨁⨁◯◯ Low |
| HbA1c | 8 | RCT | not serious | serious^f^ | not serious | serious^b^ | none | 419 | 233 | SMD **0.52 lower** (0.93 lower to 0.1 lower) | ⨁⨁◯◯ Low |
| Fasting blood glucose | 9 | RCT | not serious | serious^f^ | not serious | serious^b^ | none | 712 | 507 | SMD **0.92 lower** (1.85 lower to 0.02 higher) | ⨁⨁◯◯ Low |
| Fasting plasma insulin | 7 | RCT | not serious | serious^f^ | not serious | serious^b^ | none | 612 | 448 | SMD **0.32 lower** (0.62 lower to 0.01 lower) | ⨁⨁◯◯ Low |
| Adiponectin | 1 | RCT | not serious | not serious | not serious | serious^b^ | none | 40 | 40 | SMD **1.39 higher** (0.9 higher to 1.89 higher) | ⨁⨁⨁◯ Moderate |
| Leptin | 1 | RCT | not serious | not serious | not serious | serious^b^ | none | 40 | 40 | SMD **1.39 higher** (0.9 higher to 1.89 higher) | ⨁⨁⨁◯ Moderate |
| TNF | 2 | randomised trials | not serious | serious^c^ | not serious | serious^b^ | none | 96 | 61 | SMD **0.55 lower** (1.56 lower to 0.46 higher) | ⨁⨁◯◯ Low |
| IL-6 | 1 | randomised trials | not serious | not serious | not serious | serious^b^ | none | 40 | 40 | MD **0.55 lower** (0.92 lower to 0.18 lower) | ⨁⨁⨁◯ Moderate |
| CRP | 1 | randomised trials | serious^a^ | not serious | not serious | serious^b^ | none | 169 | 166 | SMD **0.46 lower** (0.68 lower to 0.25 lower) | ⨁⨁◯◯ Low |

*CI: confidence interval, MD: mean difference, SMD: standardized mean difference, RCT: randomized control trials,* a: Participants were not blinded, b: the included studies recorded a small sample size for both the control and intervention groups, C: there is considerable heterogeneity in the studies, d: there is moderate heterogeneity in the studies, e: “there were fewer women (10/32 (32%)) than men (22/32 (69%)) in the control group compared with the intervention group” f: there is substantial heterogeneity in the studies


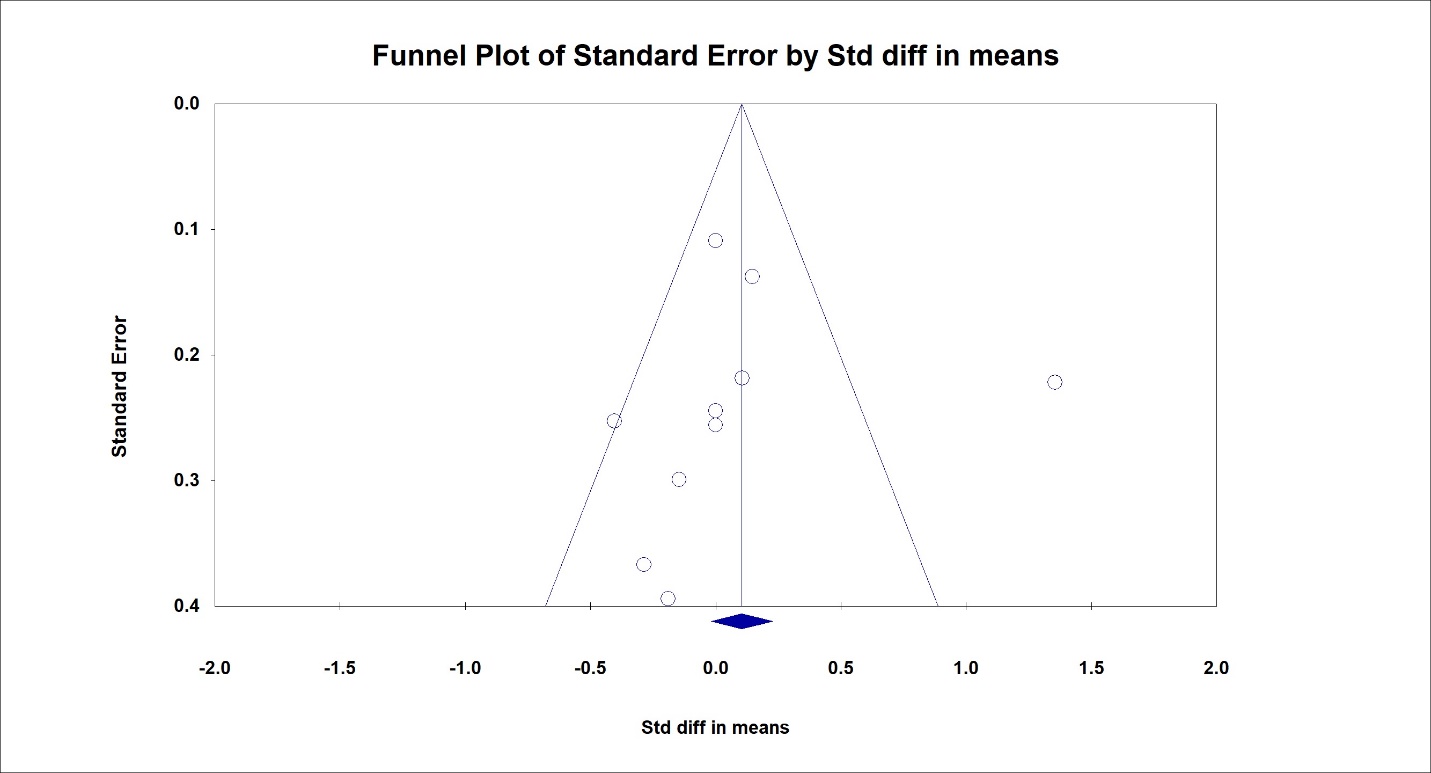
**Figure S1.** Forest plot of the effects of AEDT on HDL-C among obese T2DM showing no significant publication bias (Egger’s p = 0.86).


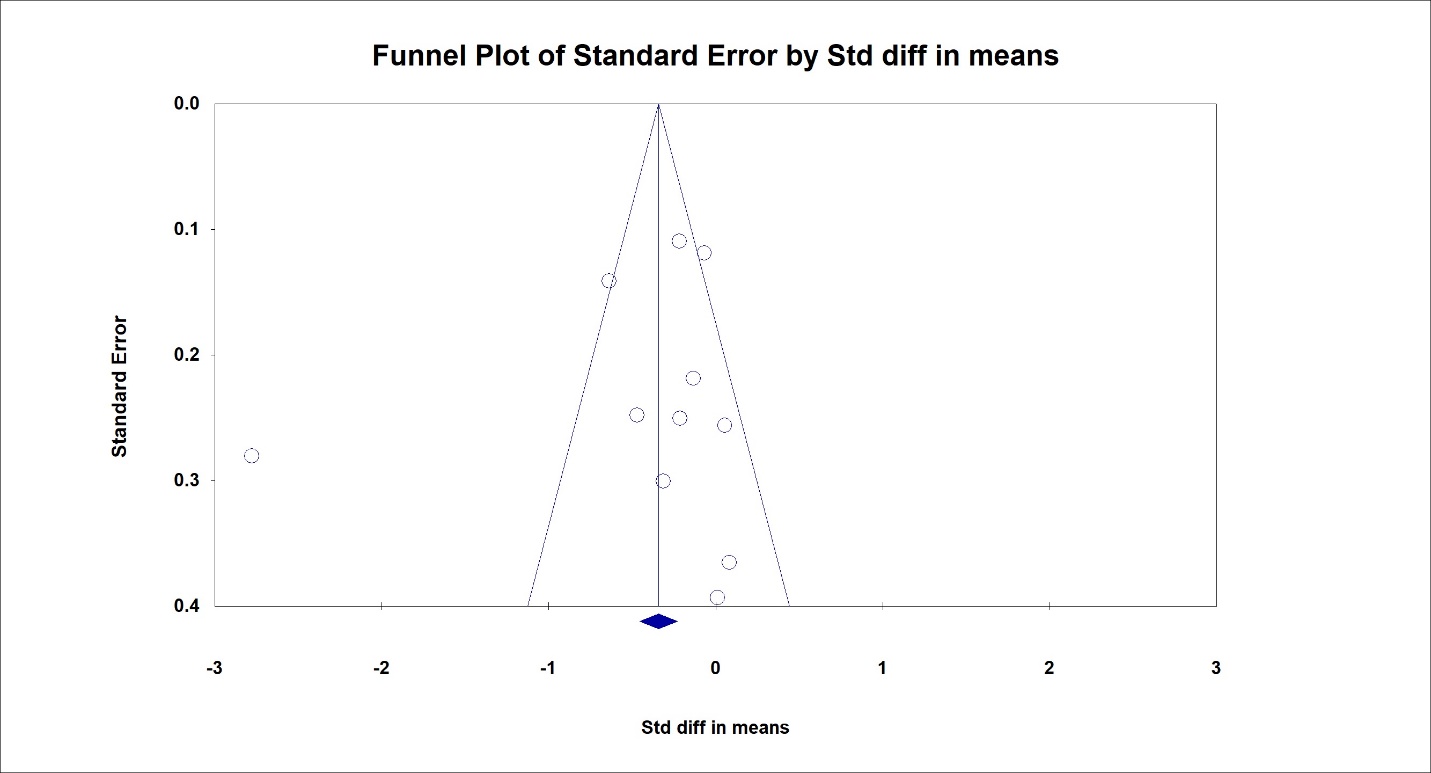
**Figure S2.** Forest plot of the effects of AEDT on TG among obese T2DM showing no significant publication bias (Egger’s p = 0.54).


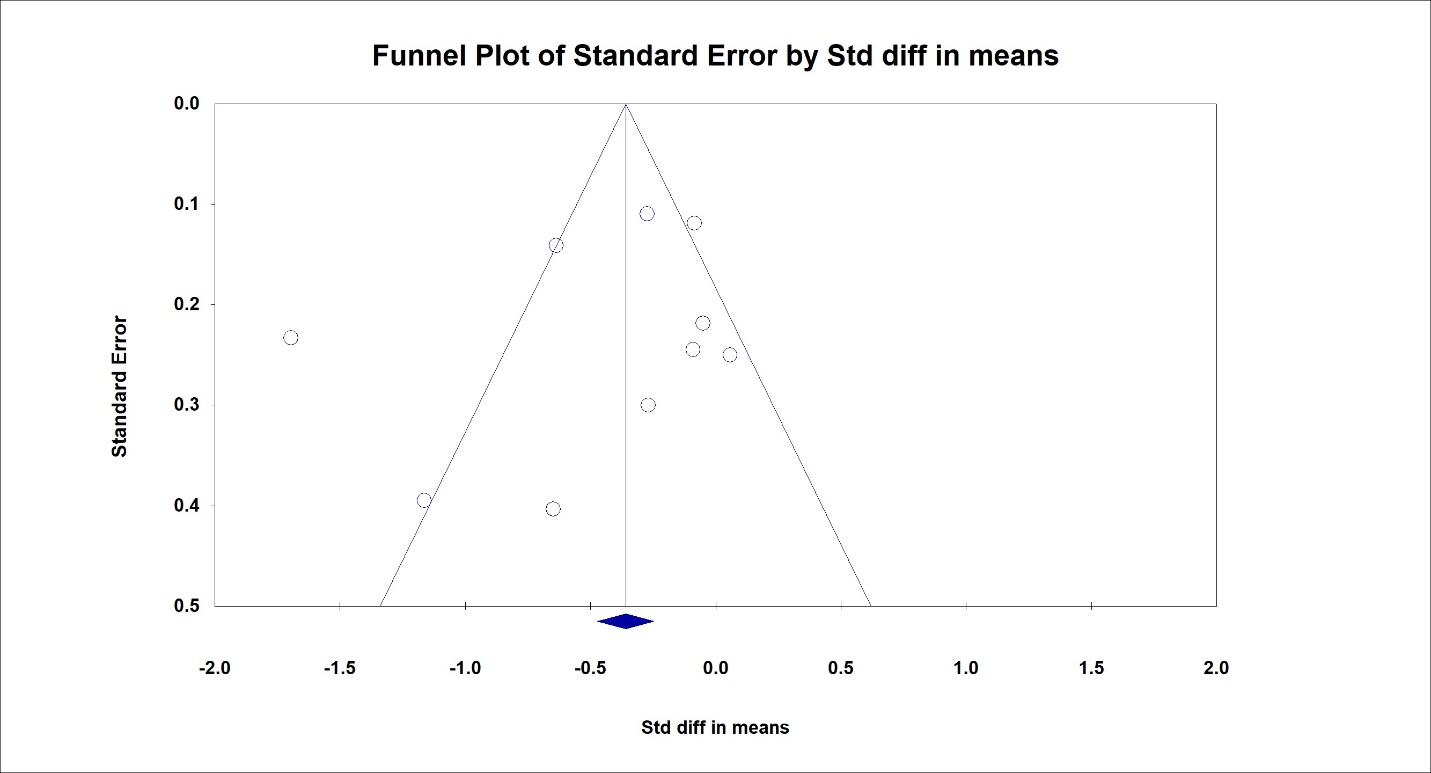
**Figure S3.** Forest plot of the effects of AEDT on TC among obese T2DM showing no significant publication bias (Egger’s p = 0.40).
